# Comparative Evaluation of Oxygen-Assisted and Compressed-Air Plasma Cutting of Low-Carbon Steel

**DOI:** 10.3390/ma19204293

**Published:** 2026-10-12

**Authors:** Anand C. Petare, Anas Ullah Khan, Sunil Dohare, Yuvraj Kumar Madhukar, Naresh Kumar Wagri, Rajesh Purohit

**Affiliations:** 1 Central Workshop, Indian Institute of Technology Indore, Indore 453552, India; 2 Department of Mechanical Engineering, Indian Institute of Technology Indore, Indore 453552, India; anasullah10@gmail.com (A.U.K.); yuvrajmadhukar@iiti.ac.in (Y.K.M.); 3 Department of Mechanical Engineering, Indian Institute of Technology Bombay, Mumbai 400076, India; 4 Department of Mechanical Engineering, University Institute of Technology, Rajiv Gandhi Proudyogiki Vishwavidyalaya (UIT-RGPV), Bhopal 462033, India; sunildohare@rgpv.ac.in; 5 Department of Materials Science and Engineering, School of Industrial Engineering and Management, KTH Royal Institute of Technology, SE-10044 Stockholm, Sweden; 6 Department of Mechanical Engineering, Maulana Azad National Institute of Technology, Bhopal 462003, India; rpurohit73@gmail.com

**Keywords:** plasma cutting, dross, kerf, Heat Affected Zone, surface roughness

## Abstract

Air plasma cutting technology is widely used for high-speed profiling of electrically conductive materials. The cut quality of surface and thermal effects is highly influenced by gas composition and process parameters. This study aims to systematically optimise and comparatively evaluate conventional compressed-air plasma cutting and an oxygen-assisted compressed-air plasma cutting process for 10 mm thick C-20 carbon steel plates. The investigation was conducted in two sequential stages using statistical Design of Experiments (DoE). First, a full-factorial design comprising 20 experiments was performed using compressed air to establish baseline process parameters and optimum operating ranges. Subsequently, a Central Composite Design (CCD) based on Response Surface Methodology (RSM) was employed for 20 additional experiments to model and compare pure compressed air with an oxygen-compressed air mixture. Cut quality was evaluated in terms of kerf width, dross height, bevel angle, surface roughness, maximum cutting temperature, and heat-affected zone (HAZ) length. The results demonstrate that the addition of oxygen improves plasma arc characteristics and produces substantial reductions in kerf width from 2.3 to 2.1 mm, dross height from 0.3 to 0.2 mm, bevel angle from 1.6 to 1.4 Radian, Avg.surface roughness from 1.2 to 0.2 µm, Max.surface roughness from 8.2 to 1.6 µm, Max. surface temperature from 1719.3 to 1607.8 °C and HAZ from 38 to 23.2 µm compared with conventional compressed-air cutting. Furthermore, microstructural analysis of the cut surfaces revealed a significant reduction in HAZ length when the oxygen-assisted gas mixture was employed. Overall, the findings demonstrate the potential of oxygen-assisted compressed-air plasma cutting to improve cutting efficiency and surface quality while reducing thermal effects, providing practical guidance for the optimisation of plasma-assisted manufacturing processes.

## 1. Introduction

**Figure 1:**
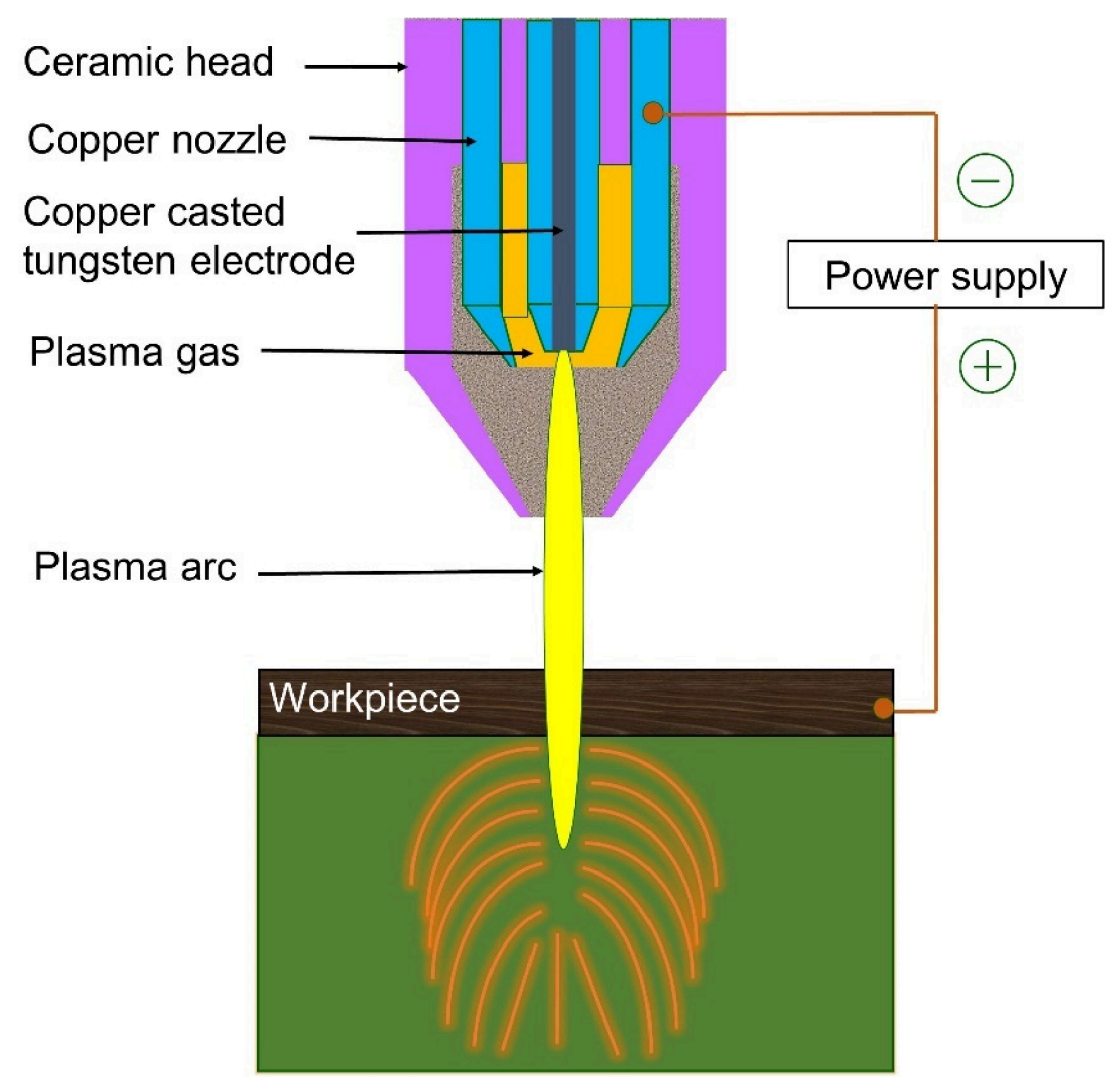
Working principle of the PAC process.

The rapid evolution of technology and increasing demands for customised shapes, complex profiles, and geometrical shapes in hard-to-machine materials have led to accentuating the use of mass metal removal processes. Laser cutting, plasma arc cutting, abrasive water jet cutting, air arc gouging and oxy-acetylene flame cutting are the most commonly used processes for cutting a variety of materials. Plasma arc cutting (PAC) is one of the non-conventional thermal cutting processes for high-quality, precise cutting of electrically conductive materials. PAC is a very fast, accurate and economical solution for straight as well as complex profile cutting of high-thickness metal plates [1,2]. Figure 1 depicts the working principle of the PAC process. In this process, an electric arc is formed between the copper-cased non-consumable tungsten electrode and the workpiece employing a high-frequency power generator. The electric arc causes the carrier gas to ionise and develops a high-speed, high-temperature plasma arc between the tungsten electrode and the workpiece. It is then blown through the fine-bore concentric copper nozzle on the surface of the workpiece [3]. The highly concentric and focused plasma energy targeted towards the workpiece surface causes localised heating, vaporisation and shearing of work materials. The carrier gas used in PAC includes compressed air, oxygen, nitrogen, hydrogen and argon individually or in their mixtures [3,4]. Out of all these gases, compressed air was most frequently used as a carrier medium in the PAC process due to its easy availability and low cost, but it may cause oxidation, which introduces porosity to the cut surface. The use of oxygen gas could enhance cutting speed, but it is expensive and requires two-stage regulators to control flow and pressure. The nozzle life is also reported to have a shorter life due to high arc-blown speed and plasma temperature [5].

The PAC process generates flame temperatures above 30,000 °C, which melts a variety of conductive materials, ranging from mild steel to high entropy alloys [5,6]. The PAC process can cut metal thicknesses ranging from 0.5 to 180 mm with close dimensional tolerances of ±3 mm and an average surface roughness ‘R_a_’ value in the range of 1.6–3.2 µm [4,7]. Plasma current, torch stand-off distance (SOD), cutting speed, gas pressure, flow rate, type and size of the nozzle, and composition of plasma gases are major process parameters of the PAC process that affect surface quality significantly [5,6,7,8]. The selection of process parameters in PAC is a very challenging and complex task to achieve a good cut surface quality. Therefore, many researchers have attempted to optimise the PAC process parameters to obtain high-quality cut surfaces and to investigate their influence on cutting a variety of materials. The highlights of their research findings, along with material applications and major outcomes, are reported in the succeeding paragraph.

Asiabanpour et al. [5] used a Taguchi orthogonal array (TOA) for optimisation of the PAC process parameters for cutting stainless steel sheets of 6 mm thickness. They reported that (a) a high cutting current causes excessive kerf width, dross formation, and high surface roughness, (b) a high air pressure could produce a rough surface with excessive splatter and bevel angle, and (c) an increase in cutting speed and torch stand-off distance results in an incomplete cut with high bevel angle and dross formation. Chen et al. [6] explored Taguchi-based Six Sigma methodology to optimise the plasma process parameters for reducing the roundness of the holes cut in 11 GA steel electrical switchboards. They reported that the TOA optimisation helped to reduce roundness error by 75% and bevel angle by 23%, respectively. Thomas [3] attempted to optimise the PAC process parameters to cut bridge structural components made of S355 steel. He reported that an increase in cutting speed significantly decreases dross formation and dragline generation. Further, the thickness of the HAZ reduced with an increase in cutting speed; however, the surface becomes brittle and harder. The surface roughness produced on the work surface was improved with cutting speed, but the bevel angle of the cut increased excessively. Salonitis et al. [7] used the PAC process for cutting S235 mild steel and AISI 304 stainless steel sheets of 15 mm thickness. They concluded that an increase in the cutting current increases HAZ, whereas a decrease in the torch stand-off distance increases bevel angle and surface roughness. Ozek et al. [8] developed a fuzzy model to evaluate surface finish for PAC of AISI 4140 steel and reported cutting speed as the most influential parameter compared to the plasma current. Celik [9] cut S235JR sheets of variable thicknesses using the PAC process and reported that a decrease in cutting current and an increase in cutting speed result in a decrease in surface roughness. Further, a decrease in cutting speed decreases HAZ. Chamarthi et al. [10] used the PAC process to cut a Hardox-400 plate of 12 mm thickness and revealed that increasing cutting voltage, plasma flow rate and cutting speed improves the cut surface quality. Maity and Baghel [11] analysed the effect of plasma arc process parameters on cut surface quality of stainless steel (AISI 316) and reported cutting speed as the most influential parameter compared to cutting current and torch stand-off distance. Adalarasan et al. [12] used TOA for optimising plasma current, cutting speed, torch stand-off distance, and gas pressure for cutting stainless steel sheets (SS-304L). They reported that a lesser plasma current and torch stand-off distance give a good-quality cut surface. Patel et al. [13] attempted machining of AISI D2 steel using PAC and reported a gas pressure of 6.5 bar, a cutting speed of 4000 m/min, and a stand-off distance of 0.5 mm as the optimum parametric combination for high-quality surface cutting of AISI D2 steel plates. Ananthakumar et al. [14] used PAC for machining of Monel 400 plates and concluded that a low value of cutting speed, gas pressure, plasma current and torch stand of distance produced an accurate and fine kerf with lesser HAZ. Gani et al. [15] employed PAC for cutting two identically assembled cold-rolled steel sheets, DC01, and revealed that the bottom surface was more heat-affected than the top surface, excluding their hardness. Further, the use of high plasma current and low gas pressure results in low deformation of the sheet. They also assessed the effects of cutting speed, gas pressure and plasma current and reported that the sheet deformation was highly influenced by the cutting current and speed, whereas the HAZ was primarily affected by cutting speed [16]. Abouzaid and Mousa [17] applied PAC for machining of copper and revealed that there was no change in its hardness and microstructure post-PAC. Lyu et al. [18] used inductively coupled plasma arc cutting for finishing of polycrystalline tin samples. They achieved requisite surface modifications in polycrystalline tin samples of variable diameters. From the above literature, it is evident that the PAC process has received significant attention in recent years owing to its many advantages and versatility. It is also evident from the literature review that there were reasonable attempts by researchers to optimise process parameters for cutting different types of materials. Compressed air consists of approximately 21% oxygen and 78% nitrogen, with small amounts of other gases. During plasma cutting, the extremely high temperatures generated within the plasma arc cause not only oxidation reactions but also significant interaction between molten metal and the surrounding nitrogen-rich atmosphere. At elevated temperatures, nitrogen interacts with the molten metal and contributes to the formation of metallic dross deposits that are comparatively more ductile and tenacious. Such dross strongly adheres to the cut edge and generally requires additional post-processing operations, such as grinding or machining, for complete removal. In oxygen-assisted plasma cutting, the additional oxygen promotes an intense exothermic oxidation reaction with iron. This reaction generates supplementary heat, enhancing material removal efficiency and increasing cutting speed. The oxidation process predominantly produces iron oxides (primarily Fe_2_O_3_), which are brittle in nature. Consequently, any oxide-based dross adhering to the cut edge can be readily removed by simple chipping or light mechanical cleaning, thereby reducing post-processing requirements [19].

However, very limited research findings are available on multi-alignment of PAC process parameters with a mixture of different carrier gases, specifically oxygen and compressed air. The effect of the maximum cutting temperature during PAC of low-carbon steel was also not studied. Very little consideration was given to the effect of cutting speed and surface roughness of the cut surface [20]. The use of oxygen gas along with compressed air is proposed to overcome the limitations of compressed air and oxygen gases while using them individually for PAC applications. This experimental research aims to investigate the effect of using oxygen and compressed air jointly as a carrier medium for cutting C-20 carbon steel. The PAC process parameters were optimised and cut surface quality was also analysed by studying kerf width, dross height, bevel (taper) angle, average surface roughness, maximum surface roughness, heat-affected zone (HAZ), maximum temperature, microstructure and microhardness.

The key novelty of this work lies in treating oxygen concentration within compressed air as a continuously controllable process parameter rather than a fixed gas composition. Unlike conventional plasma cutting studies that employ either standard compressed air or pure oxygen, the present research utilises independent mass flow controllers to precisely regulate oxygen enrichment levels in the plasma gas stream. Through a Central Composite Design (CCD)-based experimental framework, the study systematically explores the transition region between air-plasma and oxygen-plasma cutting, revealing previously unreported non-linear interactions among oxygen concentration, cutting speed, and cutting current. The optimisation provides a scientific basis for achieving an optimal balance between cut quality, process efficiency, and operating cost, thereby establishing a new process-control strategy that bridges the gap between conventional air and oxygen plasma cutting technologies.

## 2. Materials and Methods

### 2.1. Materials

In this experimental work, a C-20 carbon steel plate of 10 mm thickness was selected as the work material. This grade of steel finds many applications in the manufacturing of various engineering components, including automobile, construction, aerospace, furniture, defence, marine, and shipbuilding industries. C-20 carbon steel was selected for this study because of its good machinability, weldability, low cost, and widespread commercial availability compared with many engineering steels [21]. The chemical composition of the C-20 low-carbon steel by wt. % is 0.18% C, 0.44% Mn, 0.11% Si, 0.033% S, 0.028% P and balance Fe [22].

### 2.2. Details of Plasma Cutting Setup

**Figure 2:**
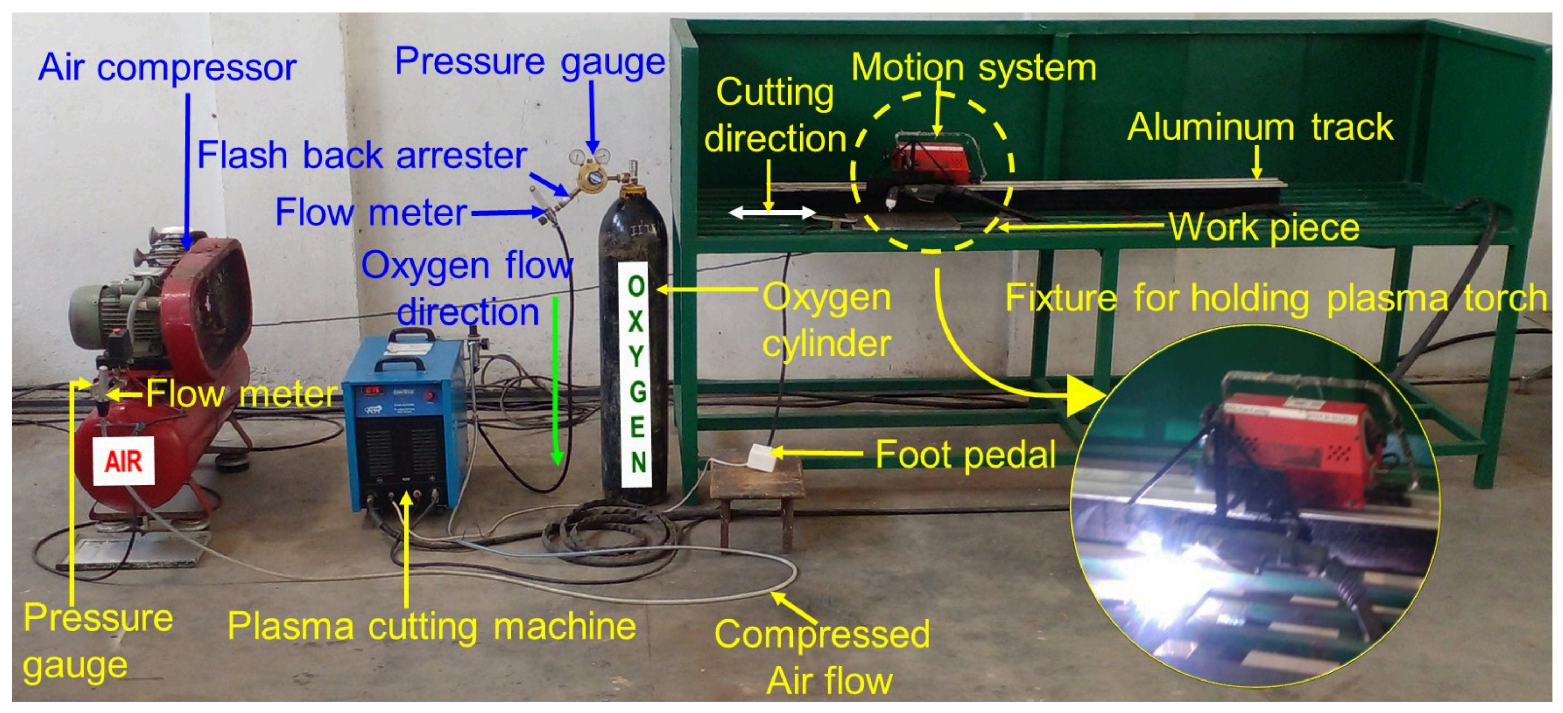
Photographs of the plasma cutting experimental setup.

All the experiments were performed on a plasma cutting machine, CWP-CUT-25 from Crux Weld, Faridabad, Haryana, India, with a dual-flow plasma torch. The machine has a facility to vary the current from 10 to 100 A, and it is connected to a compressed air tank. Figure 2 presents the photograph of the experimental setup used for the plasma cutting experiments. A unidirectional motion system (rail wheel mechanism typically employed for PUG (oxy fuel) cutting (Leopard, Esab India Limited, Ambattur, Chennai, India)) has been coupled with the plasma torch to sequentially provide automated motion in the given direction of cut. It has a facility to vary the cutting speed from 0.1 to 1 m/min and vary the torch distance up to 30 mm across the work surface from the motion system. The provided aluminium track (rail) helps to move the torch in a straight direction. The plasma torch was connected to a foot-operated switch to ON/OFF the plasma torch ensuring a safe distance of 3 m. To use a mixture of compressed air and oxygen, a T-type connector was introduced at the inlet of the PAC machine; the other two ends were connected to an oxygen cylinder and an air compressor, respectively, using hose pipes. Two pressure gauges and flow meters were mounted to the outlet of the oxygen cylinder as well as the air compressor to monitor pressure and flow rate. The flashback arrestors were installed at the inlet of the oxygen and compressed air supply for safety to trap any high-temperature particles and avoid the backflow of the respective gases. The plasma torch has a tilted neck; therefore, a dedicated attachment of a slotted bar was prepared to accommodate it on the motion system, with the arrangement to adjust the torch stand-off distance.

### 2.3. Details of Experimentation

Cutting speed, torch stand-off distance, plasma cutting current, cutting nozzle orifice diameter, type of gas used, compressed air pressure, and flow rate are dominant plasma cutting process parameters that have a significant impact on dross height, kerf width, bevel angle, and cut surface finish of work materials. A total of thirty-four preliminary experiments were performed to identify feasible ranges of cutting current ‘I’, compressed air pressure ‘P’, cutting speed ‘V’ and torch stand-off distance ‘d’ using a one-factor time approach. Therefore, plasma current varied at nine levels (i.e., 10, 20, 30, 40, 50, 60, 70, 80 and 90 A), compressed air pressure at ten levels (i.e., 1, 2, 3, 4, 5, 6, 7, 8, 9 and 10 bar), cutting speed at ten levels (i.e., 0.1, 0.2, 0.3, 0.4, 0.5, 0.6, 0.7, 0.8, 0.9 and 1 m/min) and torch stand-off distance at five levels (i.e., 1, 2, 3, 4 and 5 mm), and changes in cut surface quality were visually examined for each input parameters. In this experiment, it was observed that no cutting happened below a plasma current of 40 A due to insufficient heat generation for melting the work surface. At 50 A of cutting current, kerf generation on the work surface started, and increasing it beyond 90 A caused excessive dross build-up and large kerf width. Therefore, the cutting current range of 50–90 A was identified as a feasible range for plasma cutting. The use of compressed air pressure up to 5 bar seems to be incapable of blowing off the molten materials from the cutting track and damages the nozzle. Using compressed air pressure at 6 bar produces a sufficient arc to cut the metal, but clogging of removed materials is observed in the cutting track. Raising the compressed air pressure up to 7 bar produces a fine kerf with less dross formation. Increasing gas pressure beyond 7 bar resulted in excessive dross build-up and wider kerf due to the removal of more materials. Consequently, the compressed air pressure of 7 bar was identified as a feasible pressure for further experiments. Similarly, increasing the cutting speed below 0.5 m/min results in frequent dropping of the plasma arc and results in an incomplete cut. The cut surface quality was found to improve between 0.5 and 0.9 m/min cutting speed; increasing it beyond this resulted in excessive dross generation and poor-quality cut. Therefore, a cutting speed of 0.5–0.8 m/min was identified as a feasible range. Also, increasing the torch stand-off distance from 1 to 3 mm results in no arc generation and damages the nozzle tip due to it welding to the work material. The plasma arc started at 4 mm of torch stand-off distance; increasing it to 9 mm resulted in an incomplete cut. Hence, 4–8 mm was identified as a feasible range of torch stand-off distance. Based on the results of the preliminary experiments, twenty experiments using the Central Composite Design (CCD) approach of response surface methodology (RSM) were designed and conducted for compressed air plasma cutting of C-20 carbon steel by varying the three most important parameters at five levels each, i.e., cutting current at 50, 60, 70, 80 and 90 A; cutting speed at 0.5, 0.6, 0.7, 0.8 and 0.9 m/min; stand-off distance at 4, 5, 6, 7, and 8 mm, keeping compressed air pressure; and cut length as constant at 7 bar and 30 mm, respectively. The plasma-cut surface with the minimum values of kerf width, dross height, bevel angle, average surface roughness, maximum surface roughness, maximum surface temperature and heat-affected zone is considered the best-cut plasma surface, and the corresponding values of variable parameters are considered an optimum parametric combination. The values of the optimum parametric combination and the corresponding values of responses for the best-cut plasma surface using compressed air are presented in Table 1.

To compare the effect of the addition of oxygen with compressed air on plasma-cut surface quality, twenty experiments (including 6 experiments for central points) using the Central Composite Design (CCD) approach of response surface methodology (RSM) were designed and conducted for oxygen-assisted air plasma cutting of C-20 carbon steel by varying three most important parameters at five levels each, i.e., cutting current at 50, 60, 70, 80, and 90 A; cutting speed at 0.5, 0.6, 0.7, 0.8 and 0.9 m/min; and oxygen gas contribution (i.e., mixture of oxygen with compressed air) for plasma cutting at 40, 50, 60, 70 and 80% while maintaining a total of constant gas pressure of 7 bar at the inlet of the plasma cutting machine. This was achieved by correspondingly decreasing compressed air pressure and increasing oxygen pressure. To achieve stable volumetric flow ratios, gases were blended using independent mass flow controllers. The term ‘oxygen gas contribution’ refers to the total volume fraction of O_2_ in the mixed gas stream, which accounts for both the pure oxygen input and the 21% O_2_ naturally present in the compressed air line. The variables and fixed process parameters used in these experiments are presented in Table 2. The plasma cutting system uses compressed air only and a mixture of oxygen and compressed air, having different material removal mechanisms and associated thermodynamics. Therefore, the process parameters were optimised independently within their respective stable operating windows. For compressed-air plasma cutting, cutting speed, arc current, and stand-off distance were adjusted to enhance melt ejection efficiency and compensate for the absence of significant chemical energy contribution. In contrast, for oxygen-assisted plasma cutting, cutting speed, gas pressure, and arc current were varied to regulate the highly reactive exothermic oxidation process and achieve optimal cutting performance. Any plasma cut work surface using the parametric combination that would simultaneously minimise the considered responses was referred to as the best-cut plasma surface using a mixture of oxygen and compressed air in the subsequent text.

### 2.4. Measurement of the Responses

**Figure 3:**
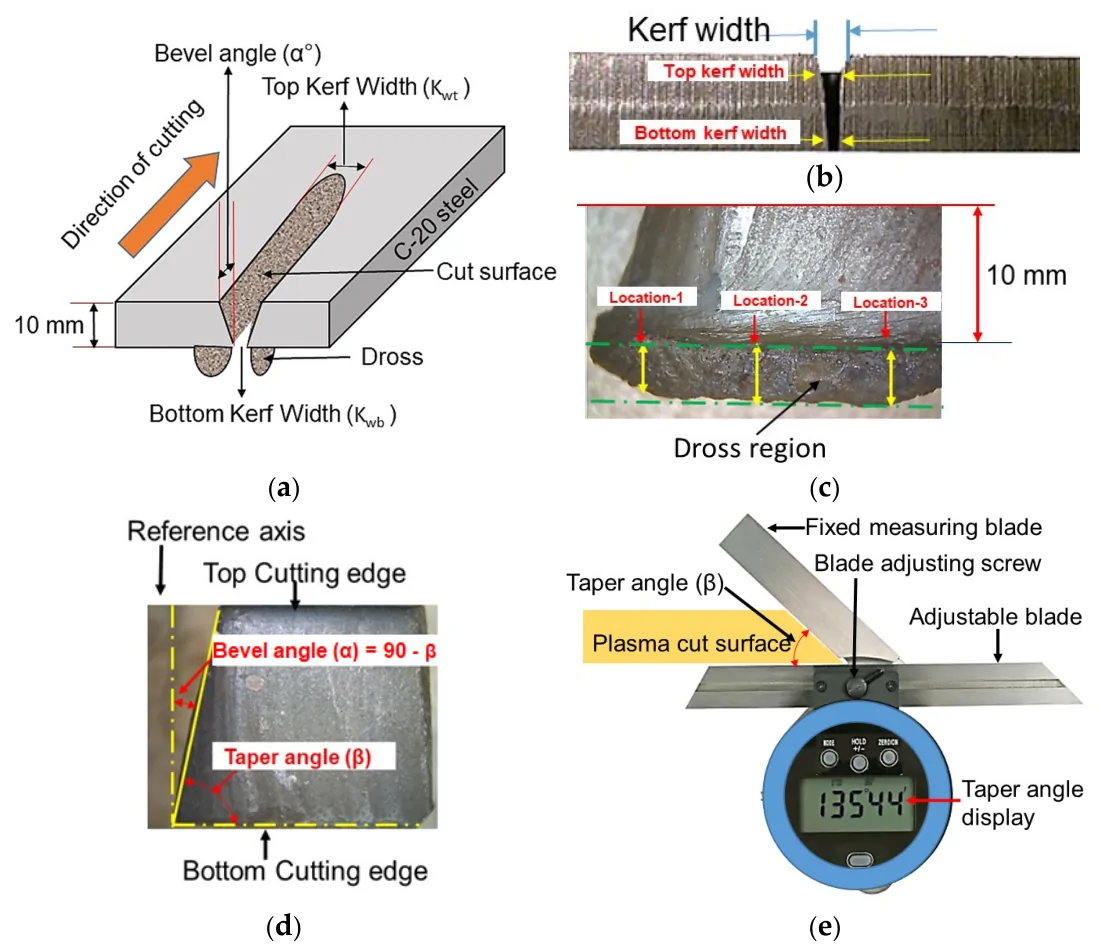
Schematics of (**a**) plasma-cut surface responses, (**b**) measurement of kerf width, (**c**) measurement of dross height, (**d**) positions of bevel angle, reference axis and cutting edges and (**e**) measurement of bevel angle.

Figure 3 depicts schematics of (a) plasma cut responses, (b) measurement of Kerf width, (c) dross height, (d) positions of bevel angle, reference axis and cutting edges and (e) bevel angle measurements. Kerf width ‘K_w_’ is the width of materials removed from the work surface during plasma cutting as it melts and blows molten metal away to create a cut gap. Kerf width ‘K_w_’ was measured using a stereo zoom optical microscope (Leica DMS1000 from Leica Microsystems, Wetzlar, Germany) and three readings at different locations from top to bottom in a kerf width (Figure 3b) of the plasma-cut workpiece sample were taken, and their arithmetic average was computed. Dross ‘d_h_’ is the height of attached solidified molten material at the bottom kerf. To evaluate the dross height, the total thickness of the plasma-cut workpiece sample was measured from top to bottom, including dross (Figure 3c), using the vernier callipers (Mitutoyo, Kawasaki, Kanagawa Prefecture, Japan) at three different locations, and the thickness of the working sample was subtracted from each value. Further, their arithmetic average was computed. The bevel angle ‘α’ is the inclination developed between the top and bottom cut edge of the plasma surface (Figure 3d). The bevel protector (Insize India LLP, Ahmedabad, India) (Figure 3e) was placed over the cutting edge to determine the taper angle (β). The taper angle (β) was subtracted from 90° to get the bevel angle (α). Further, the bevel angle (α) was converted into radians (Figure 3d).

**Figure 4:**
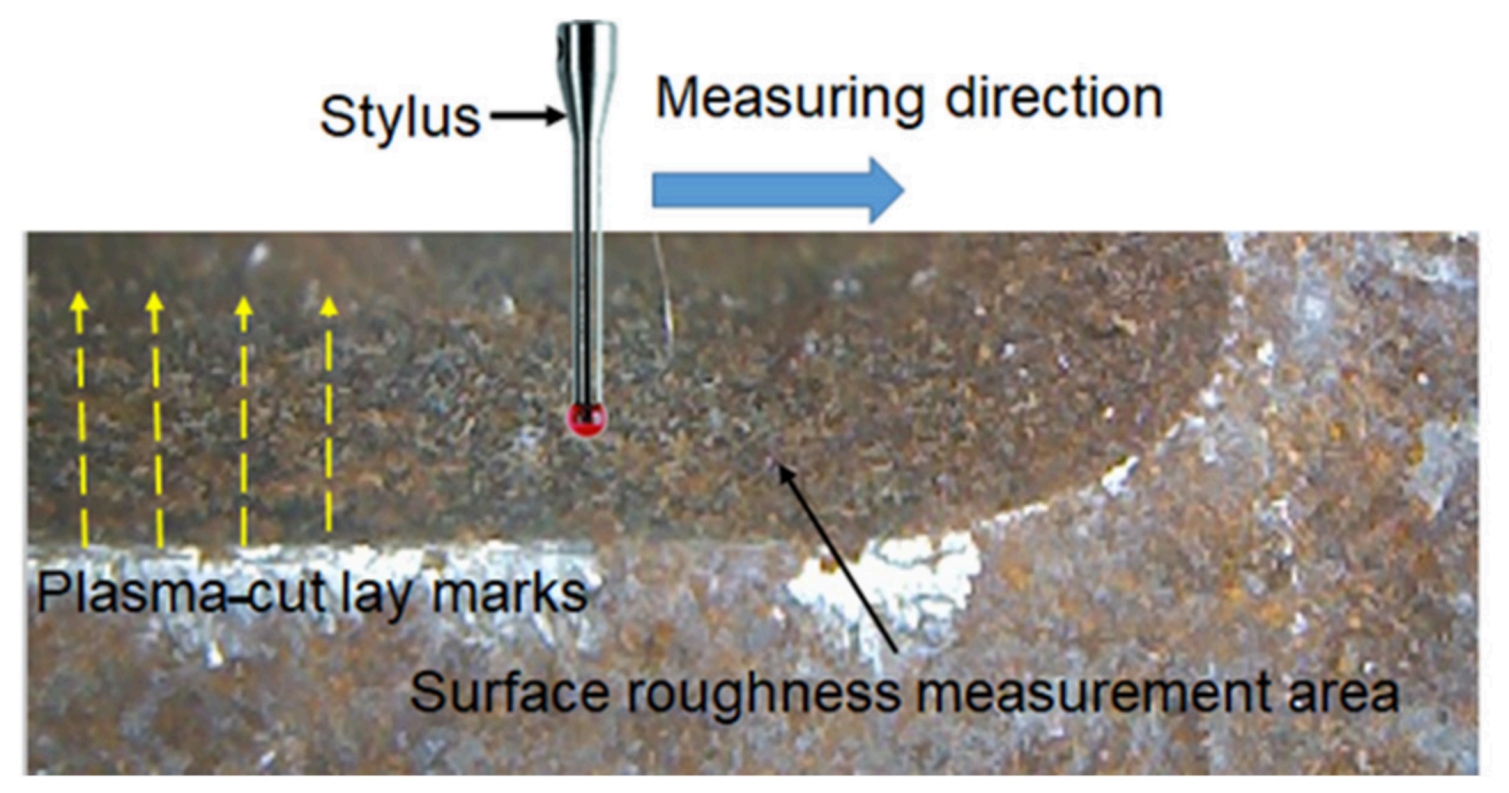
Measurement of surface roughness on the plasma-cut surface.

The surface roughness produced on the cut surface after plasma cutting depends on the selected process parameters. The maximum surface roughness ‘R_max_’ and average surface roughness ‘R_a_’ of the cut surface were measured using a surface roughness tester (ISR-C002 from Insize Co., Ltd., Ahmedabad, Gujrat, India) by tracing a 5 µm diameter stylus for a cut-off length of 0.25 mm, 1.25 mm as the evaluation length, and a Gaussian filter to distinguish between waviness and roughness parameters. To evaluate the surface roughness parameters, the workpiece sample was cut into two parts along the kerf width. The stylus probe of the roughness tester was traced perpendicular to the plasma-cut surface. Figure 4 presents a scheme of surface roughness measurement of the plasma-cut surface. Three traces at different places on the plasma-cut surface were taken, and their arithmetic average was computed for further evaluation.

The maximum surface temperature ‘T_s_’ generated during plasma cutting was recorded using a ratio pyrometer (IGAR-6 Advanced, LumaSense Technologies, Santa Clara, CA, USA). The pyrometer was installed at a 3 m distance from the plasma-cut surface to record the cut-surface temperature profile. The cooling rate was also calculated by taking the difference between the maximum temperature and the minimum measured temperature (250 °C) with respect to time duration.

**Figure 5:**
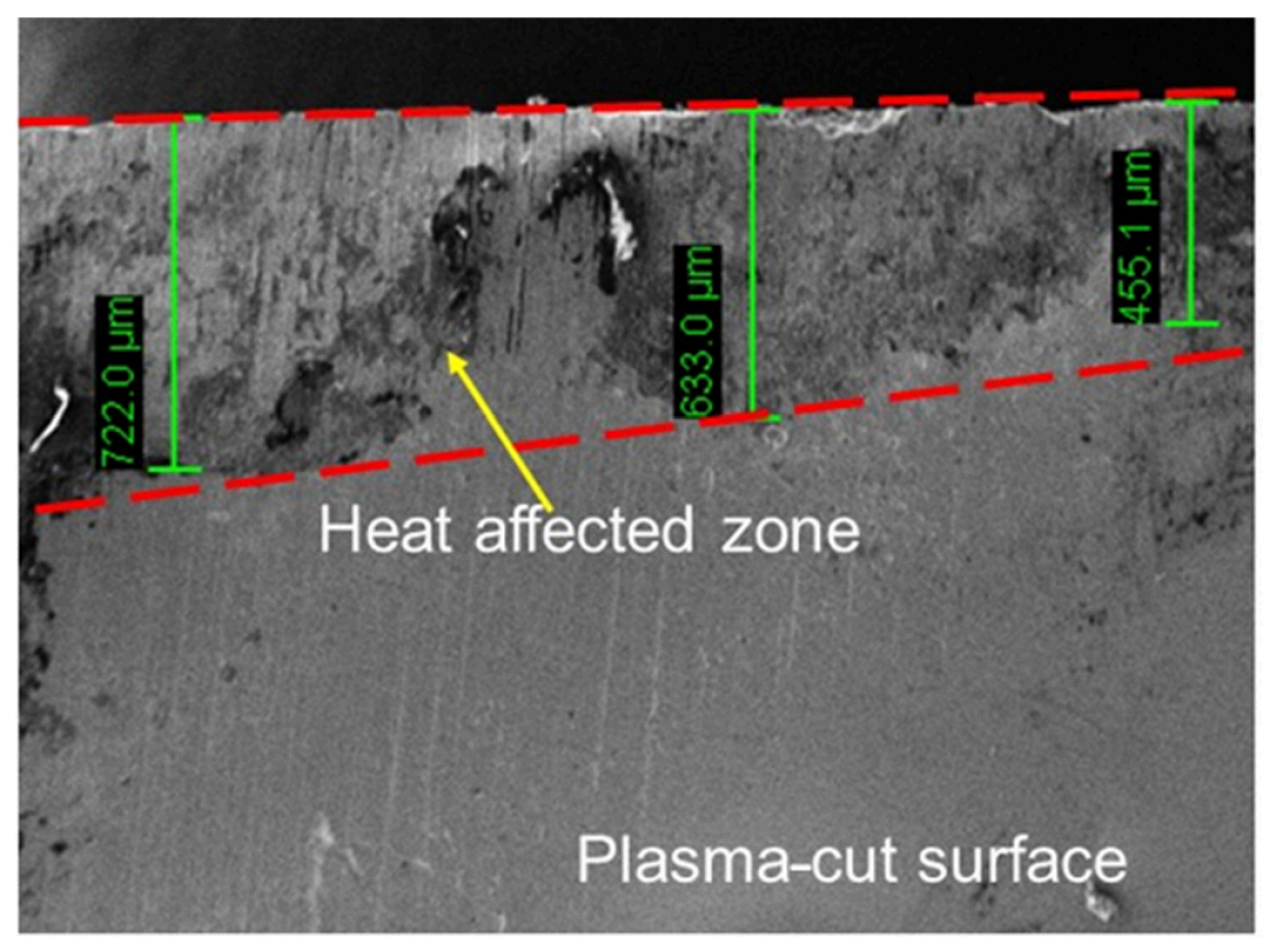
Measurement of the heat-affected zone on the plasma-cut surface.

The length of the heat-affected zone, ‘H_z_’, was measured from the edge of the cut surface. To evaluate the heat-affected zone, the samples were cut, and subsequently, a standard polishing and etching procedure was followed. Thereafter, an optical microscope (Leica DM2500M from Leica Microsystems, Wetzlar, Germany) was used to measure the length of the heat-affected zone for all the samples. Figure 5 presents measurements of the heat-affected zone. To assess the morphology and mechanism of the best-cut plasma surfaces, surface micrographs were also acquired by using an optical microscope.

**Figure 6:**
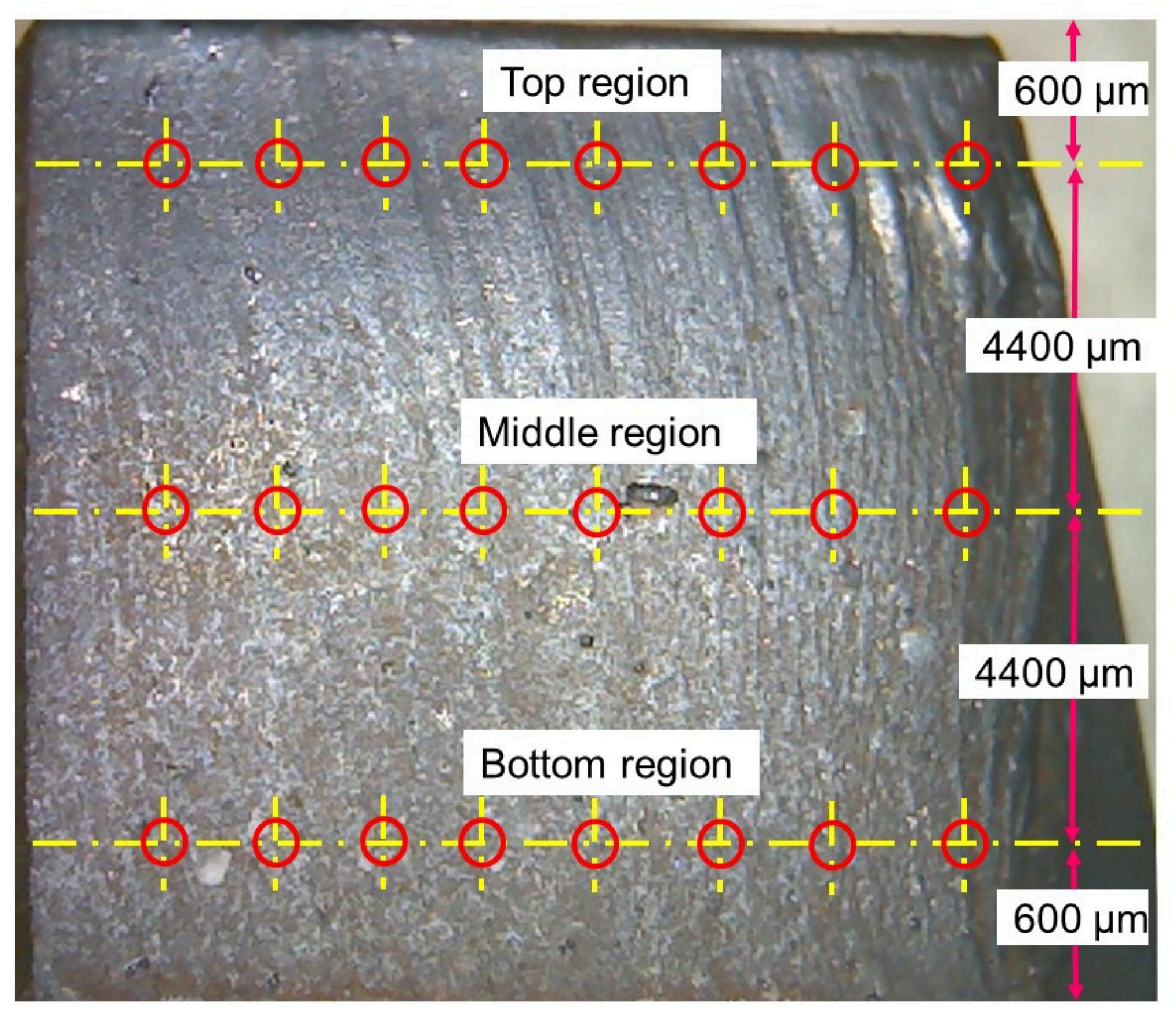
Scheme of indentation in red circle for evaluation of microhardness.

The microstructure of the best-quality plasma-cut surfaces produced using compressed air and a mixture of oxygen and compressed air was examined using a field-emission scanning electron microscope (FESEM, Supra 55, Carl Zeiss NTS GmbH, Oberkochen, Germany) to investigate the phase characteristics and surface morphology resulting from the plasma cutting process. Microhardness of the plasma-cut surfaces was measured using the Vickers microhardness tester HM-210 from Mitutoyo, Kawasaki, Kanagawa Prefecture, Japan. Indentations using a load of 100 (gm) for 15 s were done at three different locations (i.e., top, middle and bottom regions) on each sample after polishing. The scheme of indentations on unpolished samples is depicted in Figure 6. An average of these values was taken to study the effect of addition on the hardness of the workpiece material.

## 3. Results and Discussion

**Figure 7:**
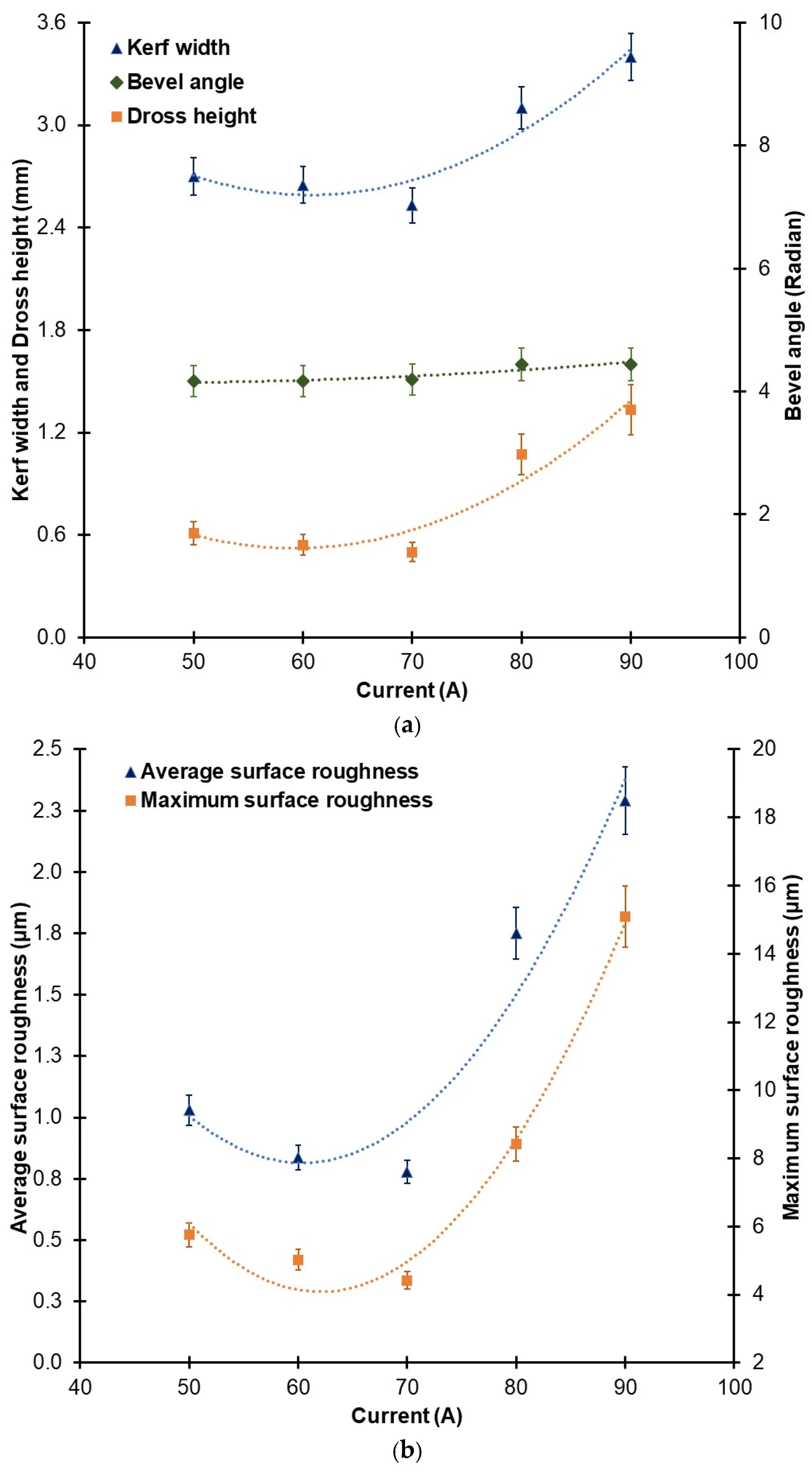

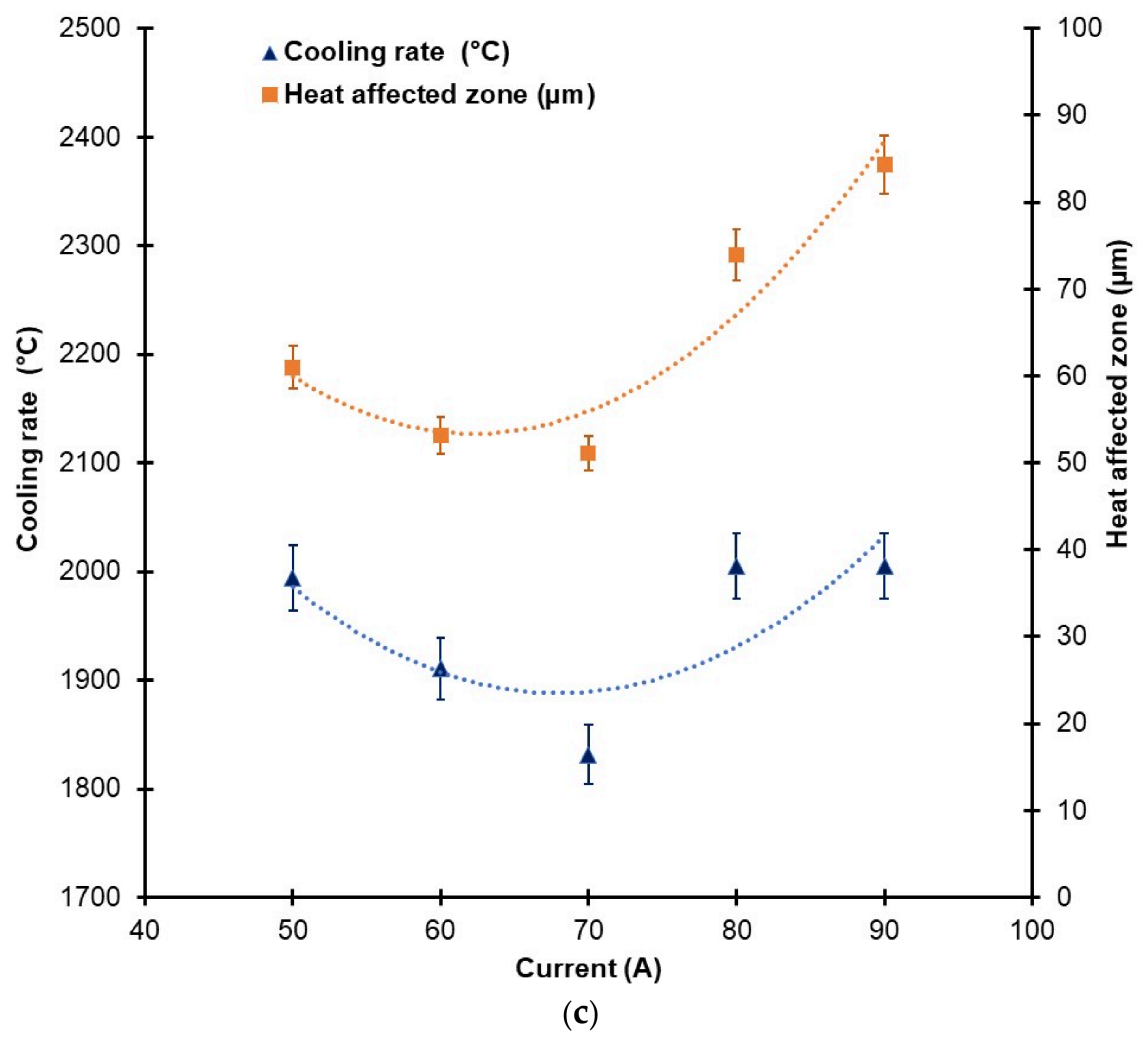
Variation of (**a**) kerf width, dross height and bevel angle, (**b**) average surface roughness and maximum surface roughness, and (**c**) cooling rate and heat-affected zone with different current values.

**Figure 8:**
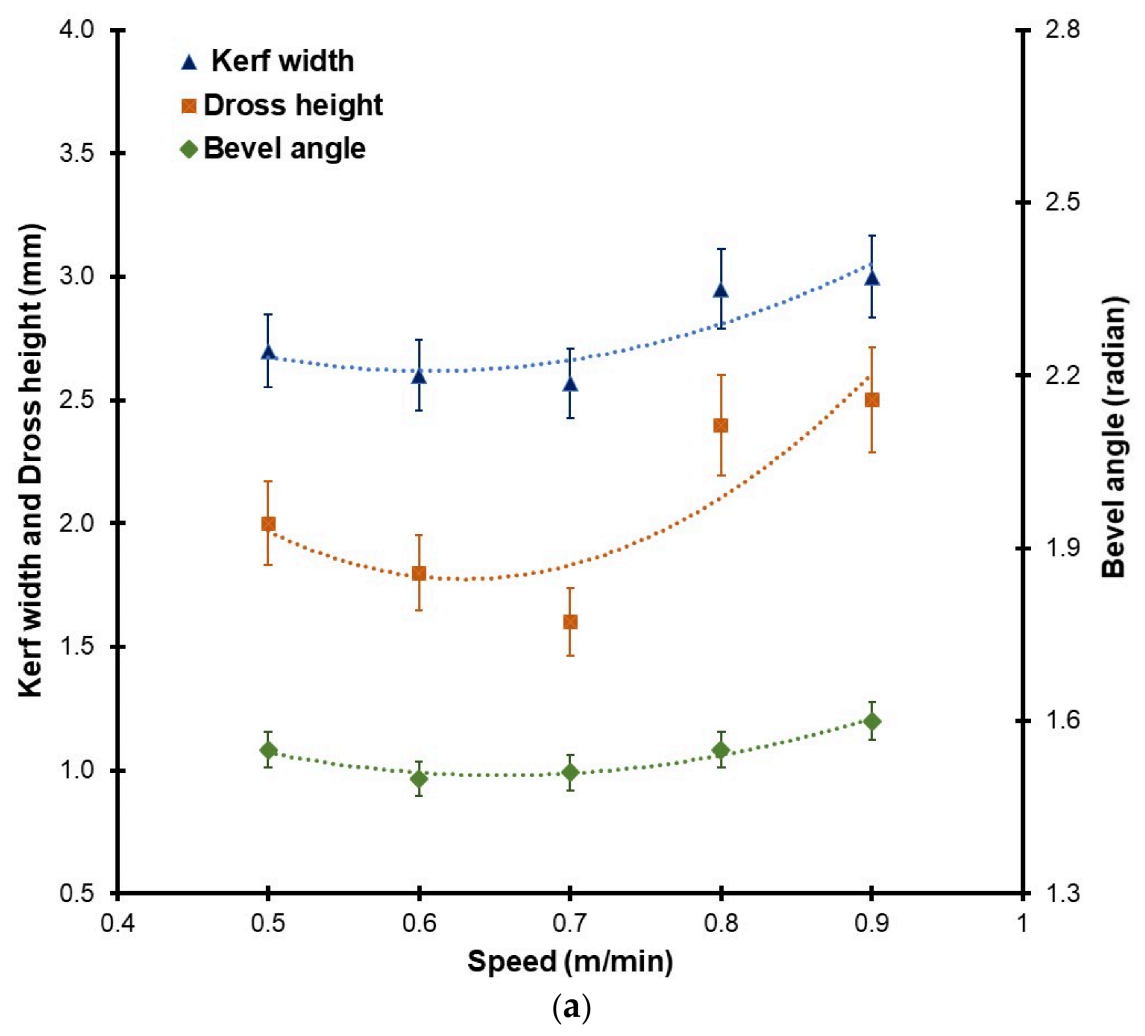

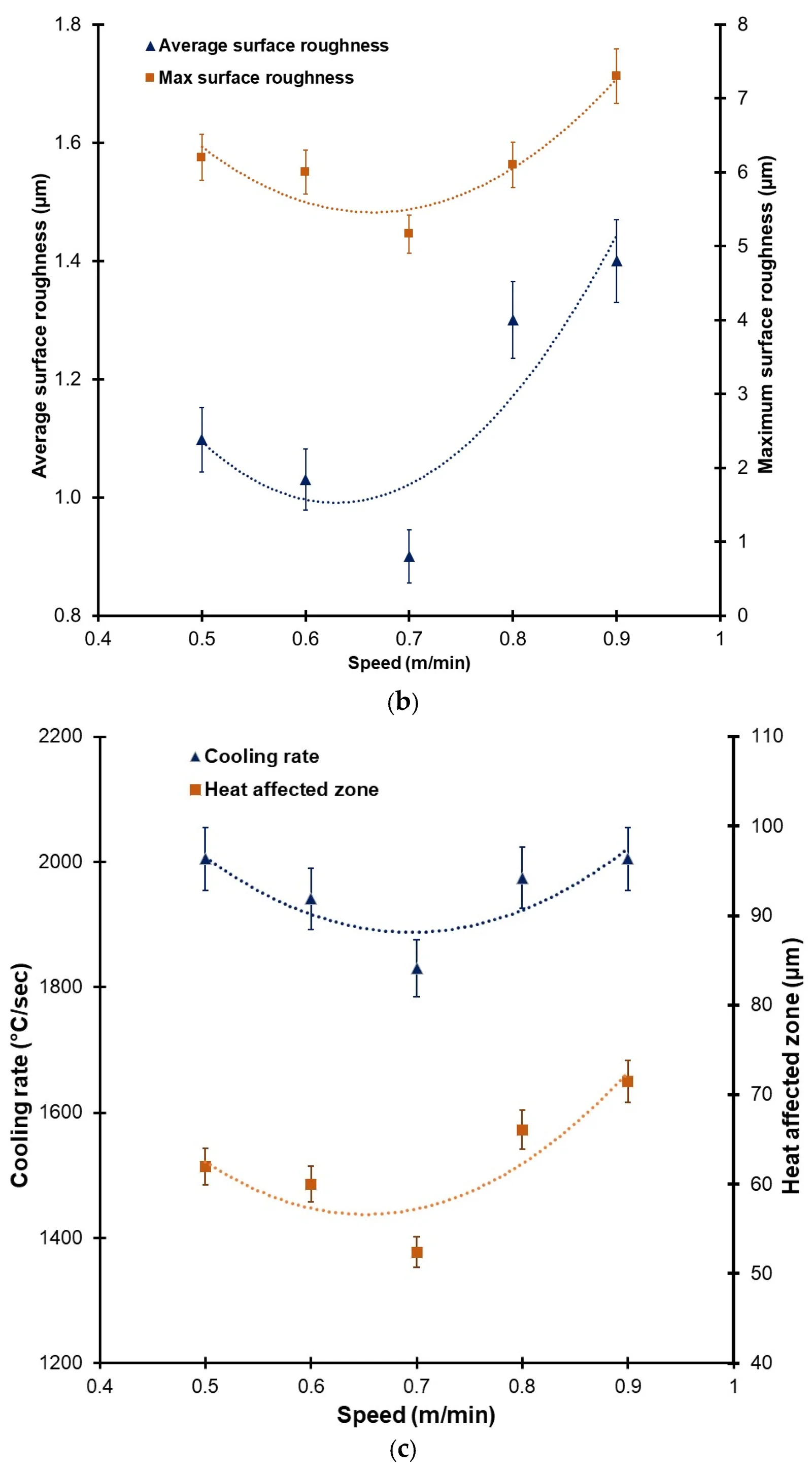
Variation of (**a**) kerf width, dross height and bevel angle, (**b**) average surface roughness and maximum surface roughness, and (**c**) cooling rate and heat-affected zone with speed.

**Figure 9:**
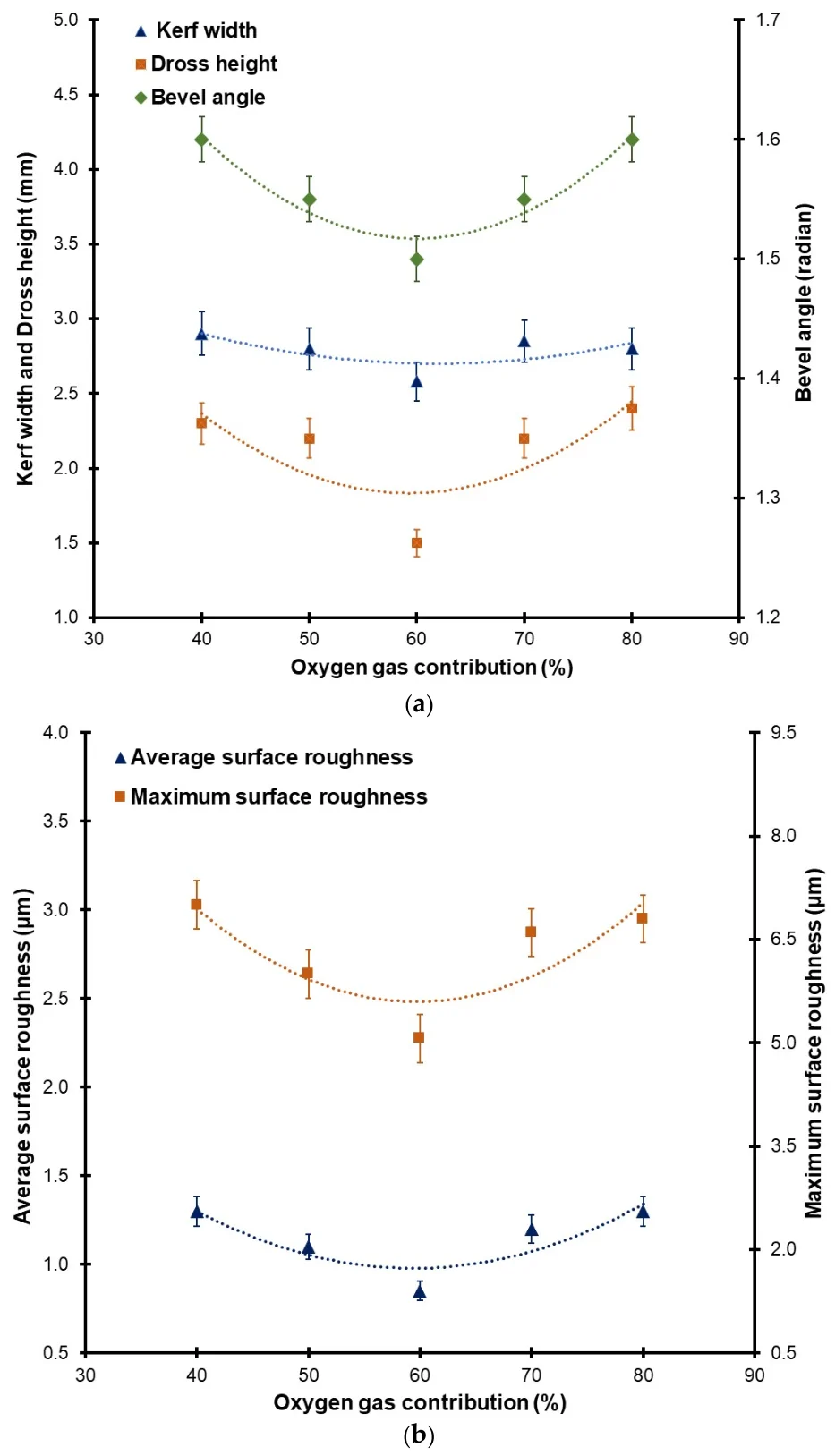

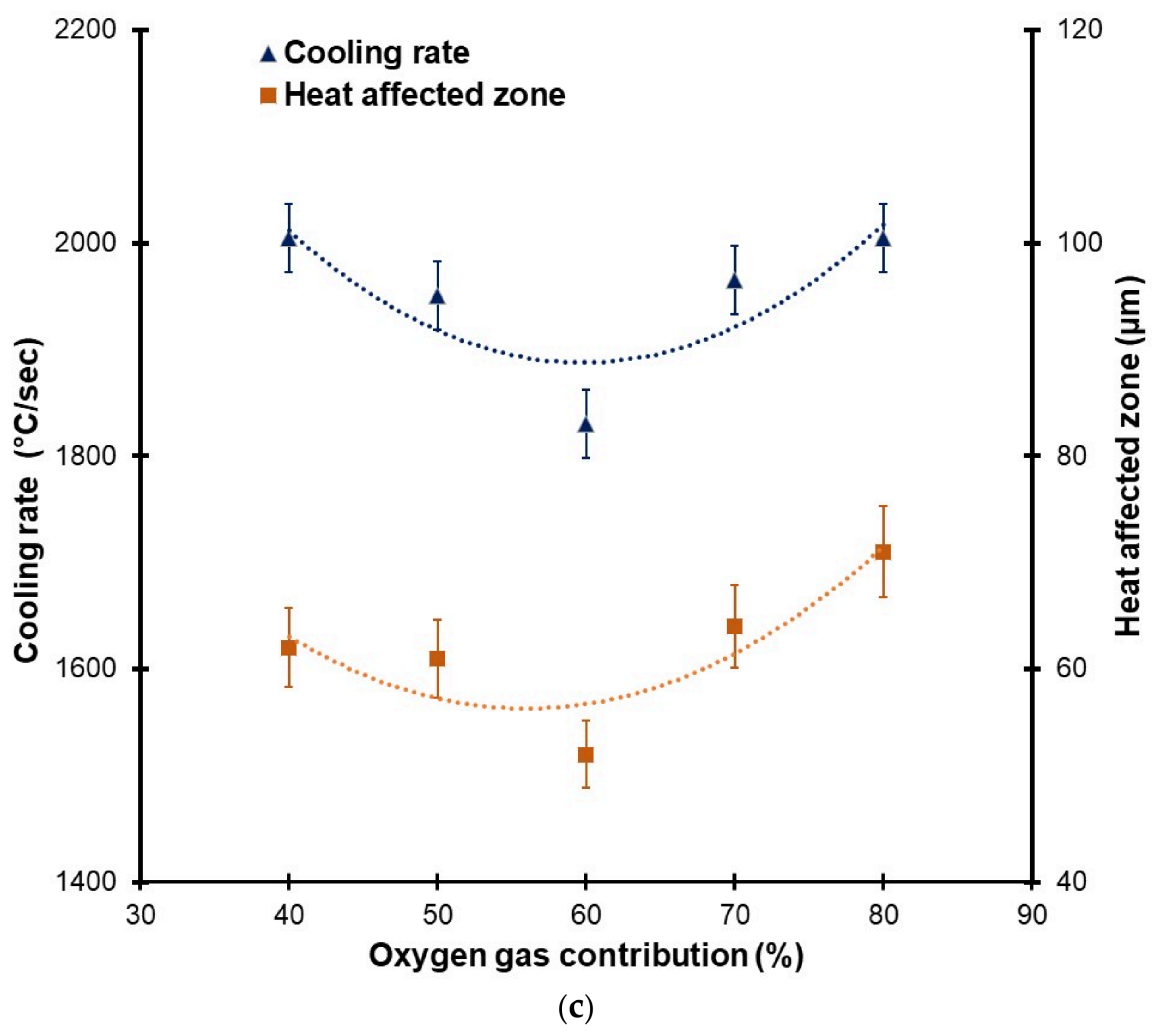
Variation of (**a**) kerf width, dross height and bevel angle, (**b**) average surface roughness and maximum surface roughness, and (**c**) cooling rate and heat-affected zone with oxygen gas concentration.

Table 3 presents responses of cut-surface quality for oxygen-assisted plasma air cutting of C-20 carbon steel for each experimental combination of cutting current, cutting speed, and gas composition. Figure 7, Figure 8 and Figure 9 present the graphical variation responses with the corresponding experimental values.

The following inferences can be made from Table 3 and Figure 7, Figure 8 and Figure 9:

- The combination of 70 A current, 0.7 m/min speed and 60% oxygen gas contribution corresponding to exp. No. 14 (Table 3) yielded minimum kerf width, dross height, bevel angle, average surface roughness, maximum surface roughness, maximum surface temperature and heat-affected zone.
- The cut quality continuously decreases with increases in the plasma current from 50 to 70 A and attains a minimum value at 70 A; thereafter, its values start increasing and attain a maximum of 90 A as depicted in Figure 7a–c. This is because less heat is available for the penetration of the work material during the initial stage. As the current value increases to 70 A, the heat content rises, and more penetration occurs, resulting in a lesser kerf width, dross height and bevel angle. This optimisation result closely aligns with the trends reported by Hussain et al. [23]. The rise in current beyond 70 A generates excessive heat, which enhances material removal and results in excessive kerf width and dross height generation with a lesser bevel angle. Similarly, during the initial stage, the values of surface roughness parameters are high due to the low current, which slows the flow of removed materials, and it solidifies quickly. It produces striations on the cut surface and raises the surface roughness values. An increase in current from 60–70 A results in an increase in heat content and uniform removal of material from the kerf, which, in contrast, reduces roughness. An increase in current beyond 70 A generates high localised heat near the kerf, and rapid removal of material takes place. This produces an uneven surface with high roughness. The slow material removal at low plasma current causes a more spread area of plasma flame over the kerf for a long time and leads to an increase in maximum surface temperature and heat-affected zone. As the current increases up to 70 A, material removal increases, the spread area of the torch reduces and the maximum surface temperature and heat-affected zone decrease. An increase in current beyond 70 A results in rapid heat cutting near the adjacent spaces of the kerf and very little time for cooling; therefore, the surface temperature rises and generates a long length of heat-affected zone.
- The cut quality decreases with an increase in cutting speed up to 0.7 m/min beyond its starting speed, increasing and attaining a maximum at 0.9 m/min, as depicted in Figure 8a–c. This is because a low cutting speed of 0.7 m/min is a strict threshold, and going below this speed increases the heat input per unit length, leading to a wider heat-affected zone (HAZ) and elevated peak temperatures. The minimisation of surface roughness ‘R_a_’ and ‘R_max_’ alongside dross height in experimental run 14 highlights the critical chemical and fluid-dynamic role of the 60% oxygen gas contribution. The presence of oxygen induces an exothermic reaction, lowering the viscosity of the molten dross and allowing the high-pressure gas jet to eject it cleanly from the kerf before it resolidifies at the bottom edge [24]. As the speed reached up to 0.7 m/min, the distribution of heat and stability of the plasma arc improved; therefore, dross generation, kerf width, bevel angle, surface roughness and heat-affected zones were reduced. An excessive rise in cutting speed up to 0.9 m/min results in incomplete penetration due to the availability of plasma arc for a very short duration, resulting in excessive dross generation with poor kerf width, bevel angle, and high surface roughness. The slow removal of dross from the cut surface again raises the heat-affected zone and cut surface temperature.
- Figure 9a–c depict the variation of kerf width, dross height, bevel angle, average surface roughness, maximum surface roughness, and heat-affected zone with oxygen gas contribution. It can be seen from these figures that the considered response parameters decrease with the rise in oxygen contribution and attain minimum values at 60% oxygen contribution. Further, a rise in oxygen contribution up to 80% again raises the response parameters. The low value of oxygen contribution generates an unstable arc and slow-blowing capacity of removed materials, which results in excessive dross formation in the cut path of the work surface. Therefore, the considered responses were high during the initial stage of the experiment as the contribution of oxygen increased up to 60%; the plasma reaction speeds up and generates a stable arc that generates a fine kerf with a lower value of dross, width and angle. The high-blown speed removed detached materials from the cut path, which reduces the values of surface roughness parameters. It also drops the cut surface temperature and reduces heat-affected zones. The rise in oxygen contribution further increases the plasma reaction rate, which results in a rapid rise in cutting temperature and results in dross generation with broad kerf width and bevel angle. The rapid removal of material generates an uneven surface, which increases the values of surface roughness parameters. The cut surface temperatures rise due to an increase in plasma reaction rate and an increase in the heat-affected zone.

Based upon the above observations, the combination of 70 A current, 0.7 m/min cutting speed and 60% oxygen gas contribution was found to be the optimum parametric combination, and the plasma-cut surface using this optimum parametric combination was referred to as the best-cut plasma surface using the mixture of oxygen and compressed air.

The regression equations were generated using experimental data listed in Table 3 in Design Expert version v25 software. In order to check the significance of each parameter on each response, an analysis of variance (ANOVA) test was performed on the experimental results at 95% confidence intervals. The ANOVA test, model significance, lack of fit, R^2^ values and regression equations in coded form are presented in Table S1 in the Supplementary Material. To identify the process parameter combination that simultaneously provides optimal cut quality, a multi-objective optimisation was performed considering minimum kerf width, minimum bevel angle, minimum dross height, minimum avg. surface roughness ‘R*_a_*’, minimum max. surface roughness ‘R_max_’, minimum surface temperature and minimum HAZ. All the process parameters have equal importance; therefore, equal weightage to all the responses was assigned. On the basis of experimental results, Multi-Objective Optimisation using Desirability Function Analysis (DFA) was performed, which predicted 63.9 A as current, 6.8 m/min as cutting speed, and 59.2% oxygen gas contribution as the optimum combination. The details of predicted values of responses are presented in Table S2 in the Supplementary Material. The cut surface quality responses for both the best-cut plasma combination (i.e., using the mixture of oxygen and compressed air and compressed air only) are compared and described in the following subsections.

### 3.1. Kerf Width, Dross Height, Bevel Angle

**Figure 10:**
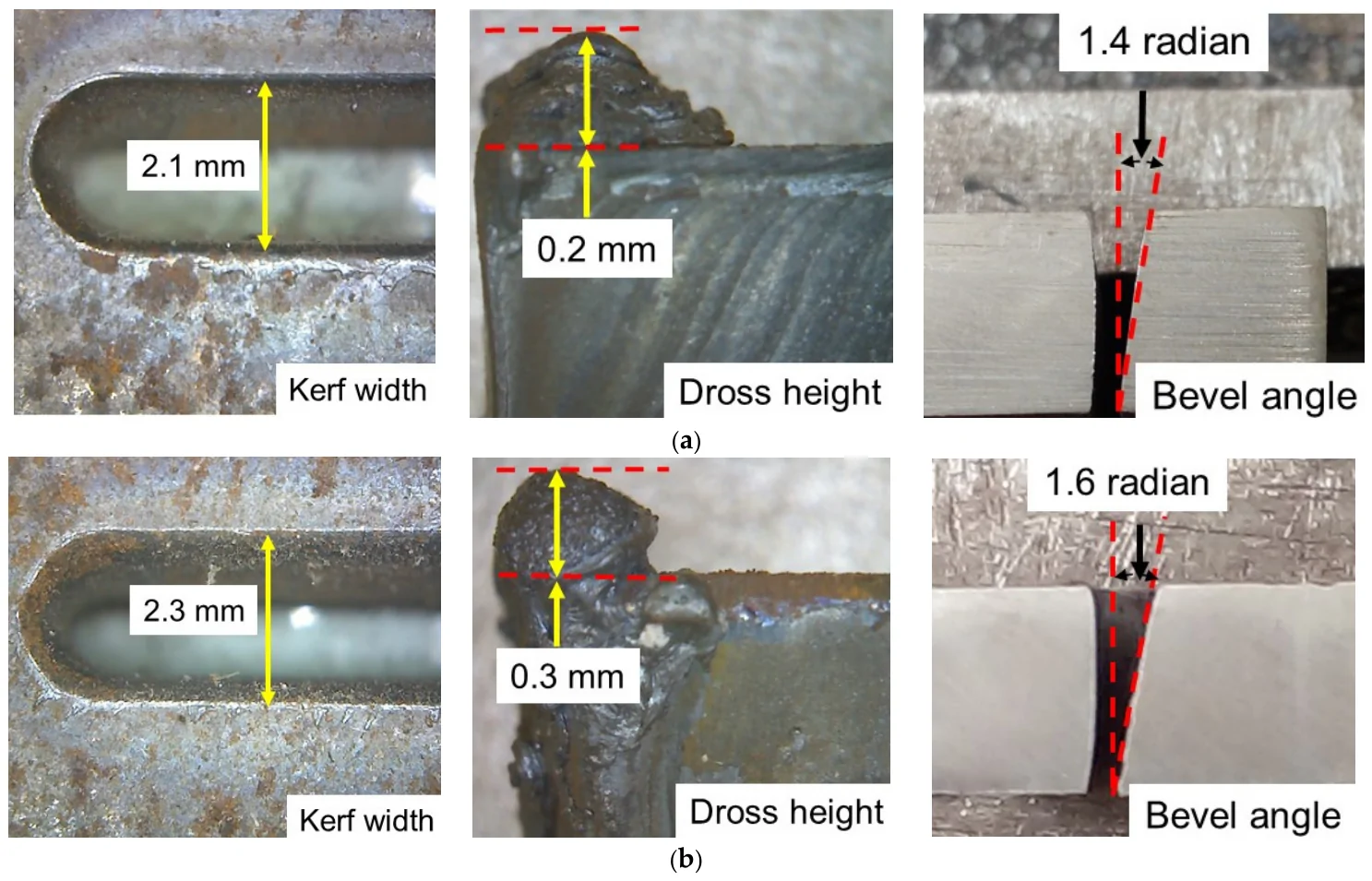
Kerf width, dross height, and bevel angle of the best-cut plasma surface using the (**a**) mixture of oxygen and compressed air and (**b**) compressed air.

Figure 10 depicts a photograph of the kerf width, dross height, and bevel angle of the best-cut plasma surface using the mixture of oxygen and compressed air and the best-cut plasma surface using compressed air, respectively. Table 4 shows the comparision of kerf width, dross height, and bevel angle of the best-cut plasma surface using the mixture of oxygen and compressed air and the best-cut plasma surface using compressed air. It can be observed from the Table 4 and Figure 10 that the values of kerf width, dross height, and bevel angle were lower in the case of the plasma-cut surface using the mixture of oxygen and compressed air compared to the plasma-cut surface using compressed air. With compressed air, the limited oxygen content results in a significant portion of the iron being removed through physical melting rather than oxidation. Molten iron possesses relatively high surface tension and viscosity, requiring substantial kinetic drag force from the plasma jet for effective ejection from the kerf. Consequently, any reduction in plasma current or variation in cutting speed can hinder molten metal removal, causing material to accumulate and solidify at the lower edge of the cut as heavy dross. As the oxygen contribution is increased, reaching an optimum level of approximately 60%, the plasma cutting process benefits from enhanced exothermic oxidation reactions. These reactions elevate the localised kerf temperature beyond that achievable through electrical energy alone, thereby improving material removal efficiency and cut quality. The additional exothermic heat generated by oxygen–iron reactions is concentrated within the cutting zone, enabling efficient material removal without requiring a broad plasma arc. As a result, the cut becomes narrower and more uniform, leading to reduced kerf width and lower bevel angle. The oxidation of iron produces iron oxide slag with significantly lower viscosity than molten iron. This low-viscosity slag is readily expelled by the plasma gas stream, minimising slag adhesion and resulting in substantially reduced dross formation along the bottom edge of the cut.

### 3.2. Surface Roughness Profiles

**Figure 11:**
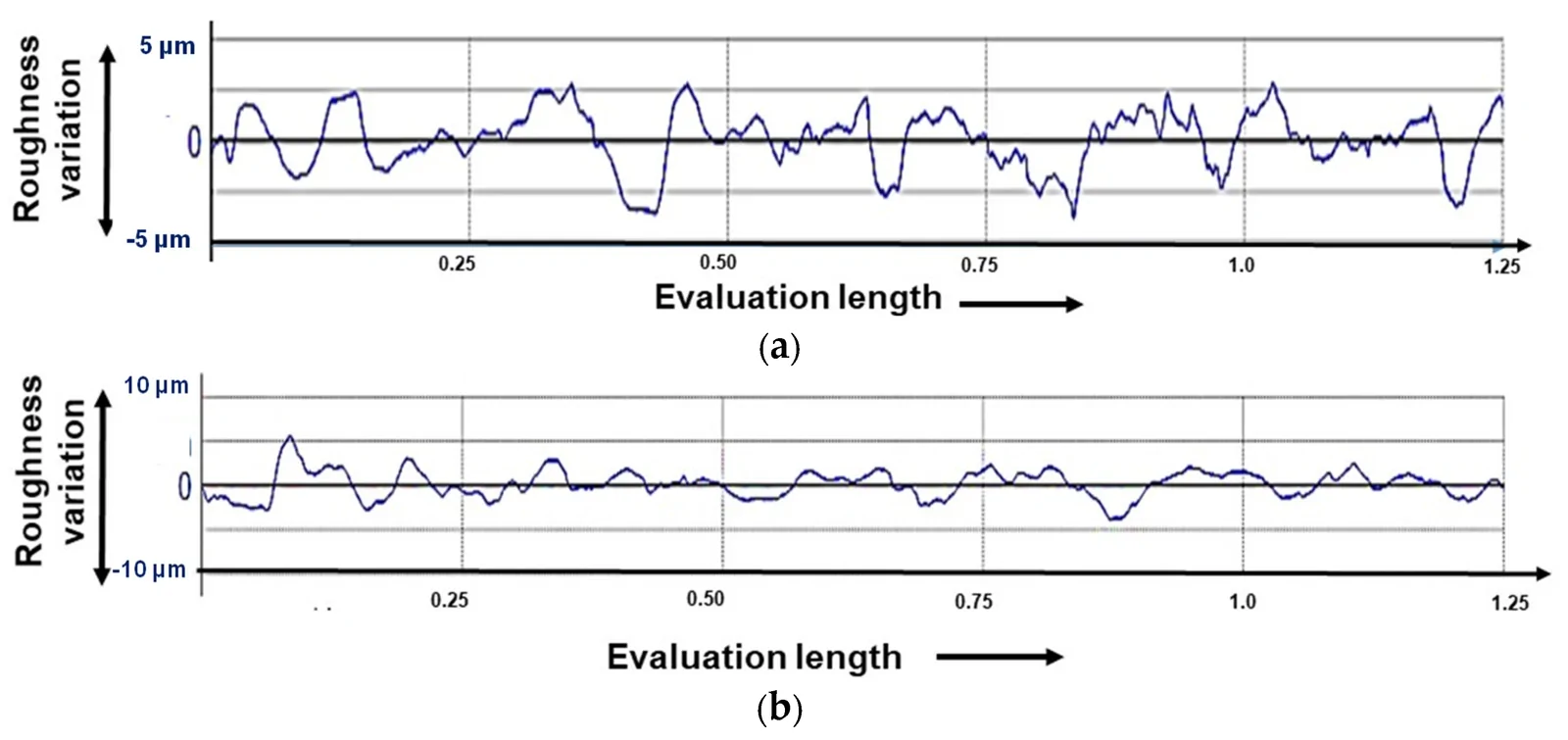
Surface roughness profiles of the best-cut plasma surface using the (**a**) mixture of oxygen and compressed air and (**b**) compressed air only.

Figure 11 depicts surface roughness profiles of the best-cut plasma surface using the mixture of oxygen and compressed air (Figure 11a) and the best-cut plasma surface using compressed air (Figure 11b), respectively. It can be observed from Figure 11 that the introduction of oxygen with compressed air improved the surface quality of the plasma-cut surface by reducing the values for avg. and max. surface roughness from 1.2 µm and 8.2 µm (i.e., plasma-cut surface with compressed air) to 0.2 µm and 1.6 µm (i.e., plasma-cut surface using oxygen and compressed air).

### 3.3. Surface Morphology

**Figure 12:**
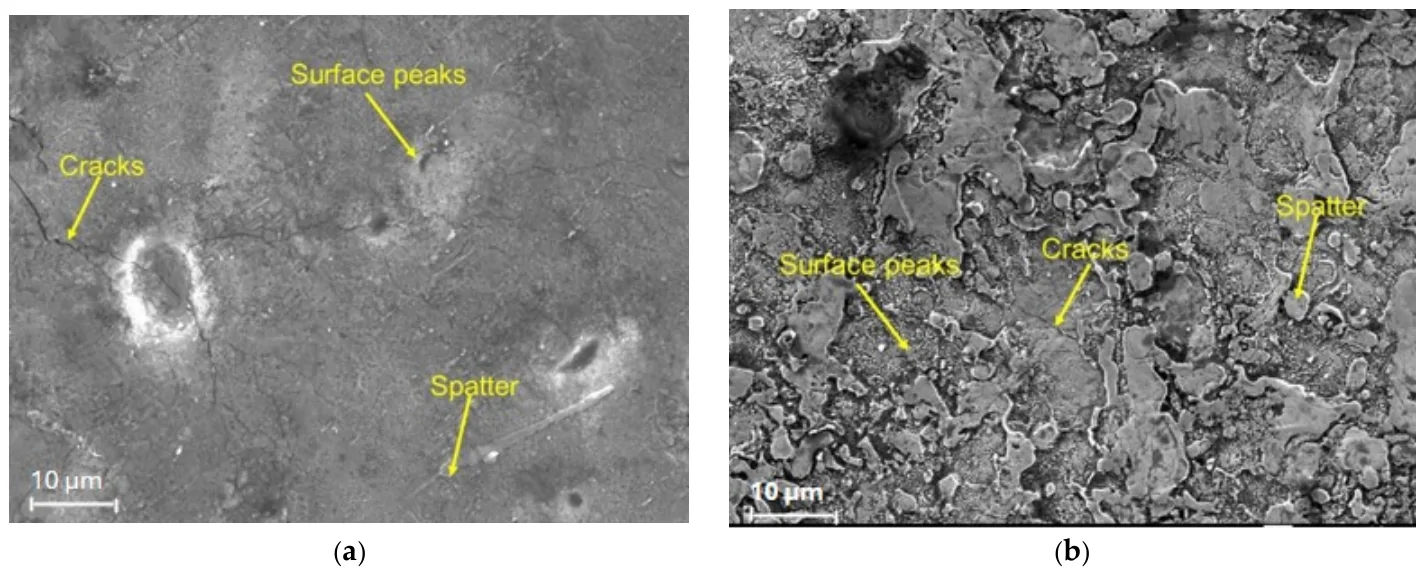
Micrographs showing the surface morphology of the best-cut plasma surface using (**a**) a mixture of oxygen and compressed air and (**b**) compressed air only.

**Figure 13:**
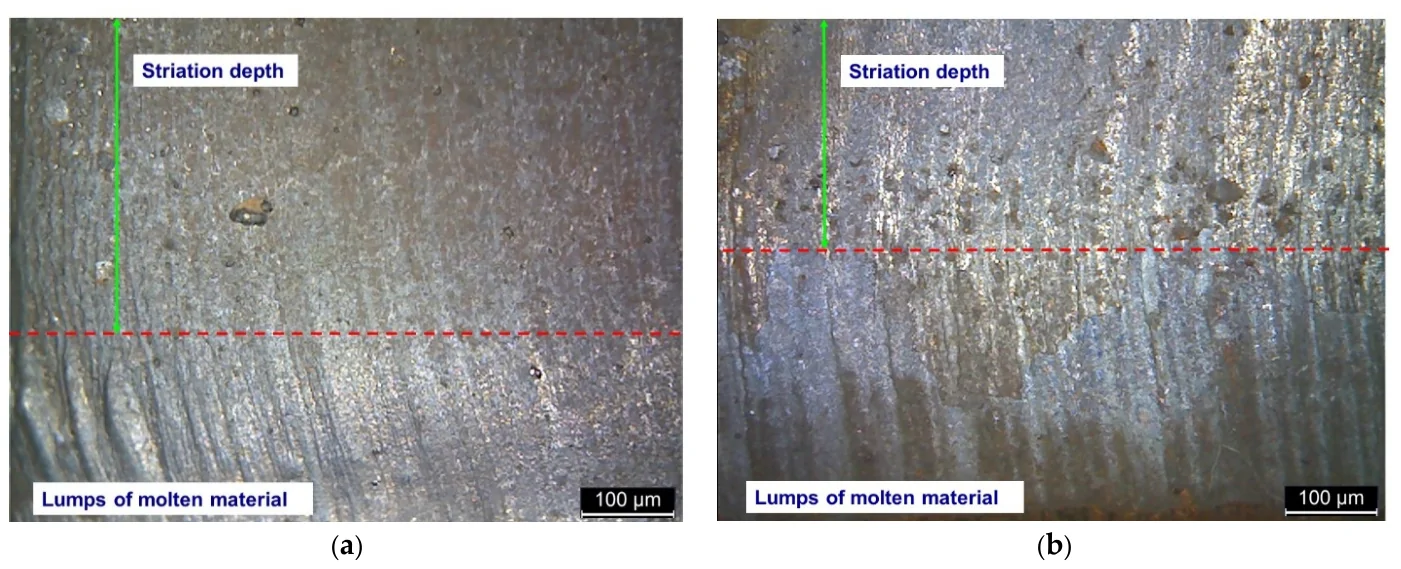
Surface patterns for the plasma-cut surface using (**a**) a mixture of oxygen and compressed air and (**b**) compressed air only.

Figure 12 depicts microscopic images showing the surface morphology of the best-cut plasma surface using the mixture of oxygen and compressed air and the best-cut plasma surface using compressed air only. It was observed that both the best-cut plasma surfaces contain surface roughness peaks, spatter, and a few cracks on the cut surface. However, it is evident that the best plasma surface using oxygen and compressed air has better surface quality than the surface quality produced by compressed air only. The plasma cutting process is considered to be a thermo-mechanical process where heating, melting, partial vaporisation and shearing/ejection of the molten material from the bottom of the kerf take place in an intense plasma arc and assist gas. In the partial vaporisation zone (depth), the material removal from a kerf typically takes place in a controlled way due to vaporisation and shearing force up to a certain depth (called striation depth), whereas the shearing forces exerted by the assist fluid become the dominant mechanism of material removal with the increase in cut depth. In general, at this depth, the flow becomes uncontrolled, and the removed materials start depositing on the wall of the cut surface, which deteriorates the cut-surface quality [25]. From Figure 13, it can be seen that the striation depth in the case of the plasma-cut surface using a mixture of oxygen and compressed air (Figure 13a) was greater than that of the plasma-cut surface using compressed air (Figure 13b). In the case of plasma cutting with compressed air, the material flow remains stable up to a limited depth; thereafter, the flow becomes unstable due to lower heat content, resulting in solidification of molten materials on the cut surface, which deteriorates cut surface quality. In the case of plasma cutting with a mixture of oxygen and compressed air, the addition of oxygen increases the heat content of the plasma due to an exothermic reaction, which prevents the solidification of the molten materials on the cut surface and improves the cut surface [26].

### 3.4. Assessment of Microhardness

**Figure 14:**
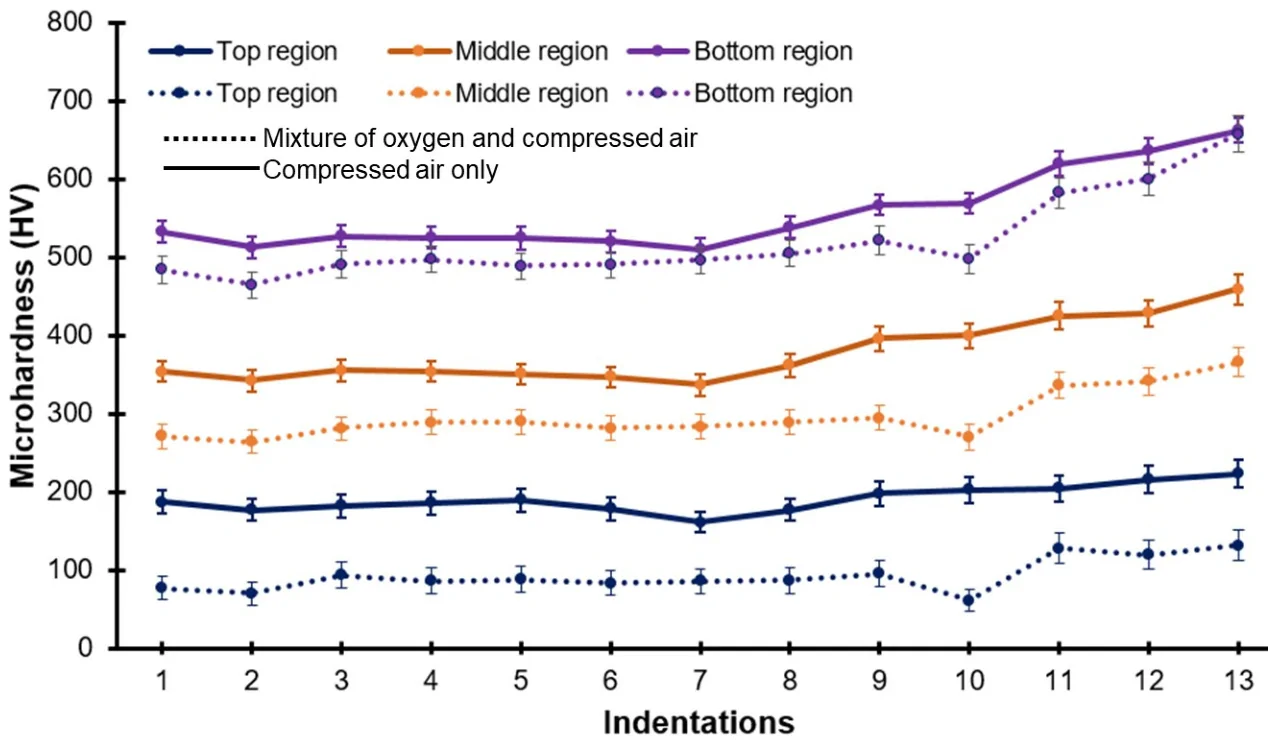
Microhardness comparison of the best-cut plasma surface using the mixture of oxygen and compressed air, and compressed air only.

Figure 14 depicts the results of the hardness assessment on the best-cut plasma surface using the mixture of oxygen and compressed air and the best-cut plasma surface using compressed air only. It is evident from the figure that the hardness of the best plasma surface using the mixture of oxygen and compressed air is lower than that of the plasma-cut surface using compressed air only. The microhardness profile obtained during compressed-air plasma cutting showed a pronounced gradient across the cut thickness. The maximum microhardness value was approximately 525 HV in the top region and gradually decreased to 210 HV near the bottom region. This behaviour can be attributed to the formation of a hardened surface layer resulting from the rapid cooling and solidification of molten metal, along with the development of the heat-affected zone (HAZ).

In contrast, plasma cutting performed using a mixture of oxygen and compressed air exhibited a more uniform microhardness distribution across the cut thickness. The microhardness values varied from 525 HV in the top region to 134 HV in the bottom region, indicating a more stable microstructural condition after cutting. The addition of oxygen promotes enhanced oxidation and heat generation during the cutting process, leading to a more uniform thermal cycle and consequently a more homogeneous microstructure within the HAZ. These results suggest that oxygen-assisted plasma cutting contributes to improved microstructural stability and reduced hardness variation across the cut section [27,28].

### 3.5. Assessment of Heat-Affected Zone

**Figure 15:**
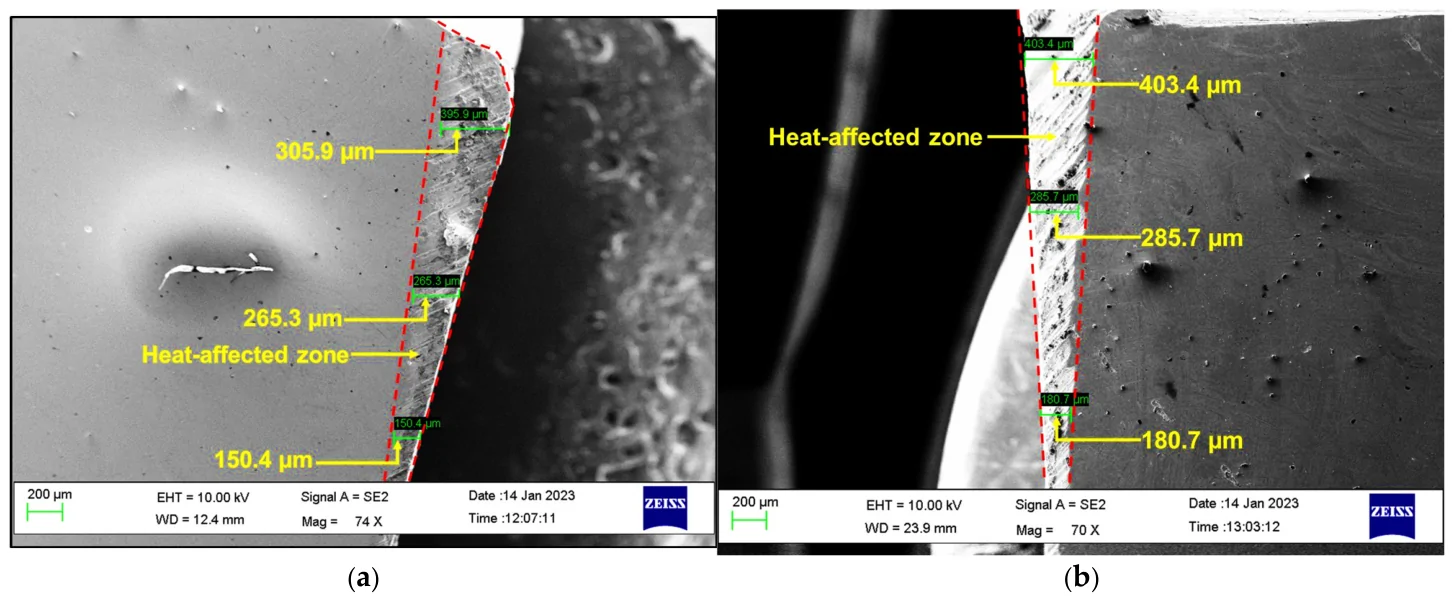
Heat-affected zone of the best-cut plasma surface using the (**a**) mixture of oxygen and compressed air and (**b**) compressed air only.

**Figure 16:**
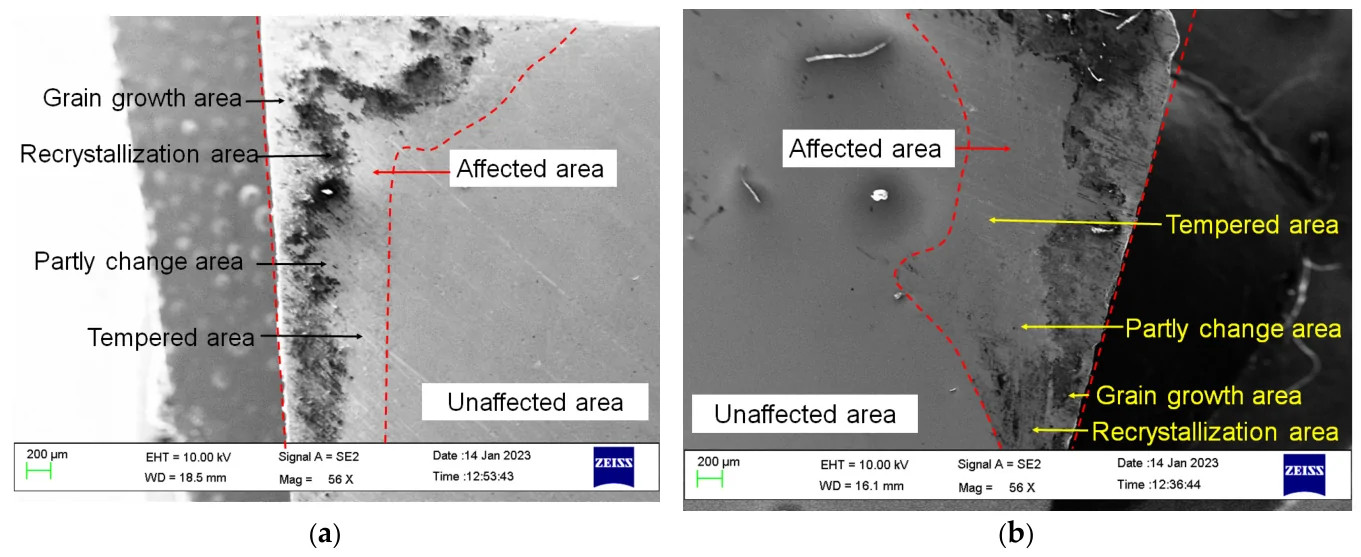
Various areas of heat-affected zones of the best-cut plasma surface using (**a**) a mixture of oxygen and compressed air and (**b**) compressed air only.

**Figure 17:**
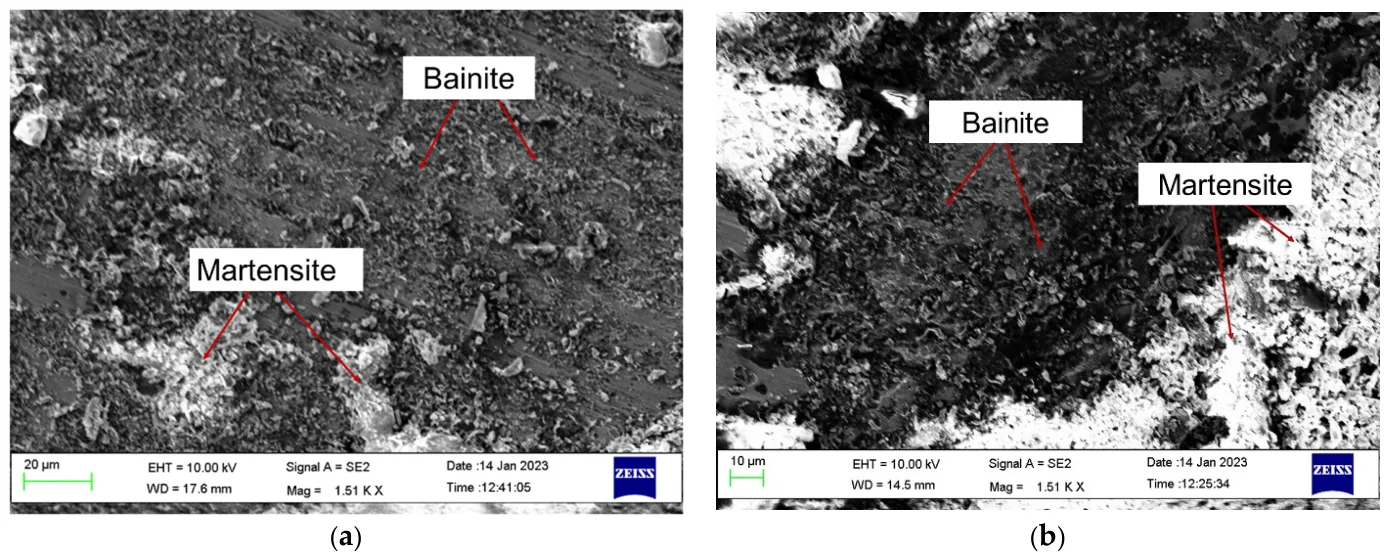
Microstructural properties of the best-cut plasma surface using the (**a**) mixture of oxygen and compressed air and (**b**) compressed air only.

Figure 15 depicts an SEM micrograph of the heat-affected zone produced on the best-cut plasma surface using the mixture of oxygen and compressed air (Figure 15a) and the best-cut plasma surface using compressed air (Figure 15b) only. It can be seen from these figures that the plasma-cut surface using the mixture of oxygen and compressed air has a shorter heat-affected zone length as compared to the plasma surface using compressed air only. The distribution areas (i.e., tempered area, partly changed area, recrystallisation area, and grain growth area) of the heat-affected zone were also low in the case of the plasma-cut surface with a mixture of oxygen and compressed air (Figure 16a) compared to the plasma-cut surface with compressed air only (Figure 16b). The addition of oxygen to compressed air enhances the energy density of the arc, stabilises it, and improves cutting. Furthermore, the increased speed of the torch leads to a shorter time for deep heating of materials, thus producing a narrower HAZ along the cutting edge. Figure 17 presents SEM micrographs of the microstructural characteristics of the best-quality plasma-cut surfaces obtained using the oxygen–compressed air mixture (Figure 17a) and compressed air alone (Figure 17b). The microstructure of both plasma surfaces contained martensite and bainite phases. Comparing both microstructures, it is clear that the area of the martensite phase is smaller in the case of plasma cutting using oxygen and compressed air than that of plasma cutting using compressed air.

The presence of oxygen during plasma cutting of work materials releases supplemental thermal energy into the cut zone, which helps to slow cooling with the associated rejection of carbon by diffusion, which reduces martensite formation and improves ductility. In contrast, using compressed air alone while plasma cutting work materials causes rapid cooling and results in a large martensite structure formation. The presence of material flow marks, dross, surface dragline and heat-affected zone indicates the melting and blasting action mechanism of the plasma jet for material removal.

### 3.6. Assessment of Maximum Surface Temperature

**Figure 18:**
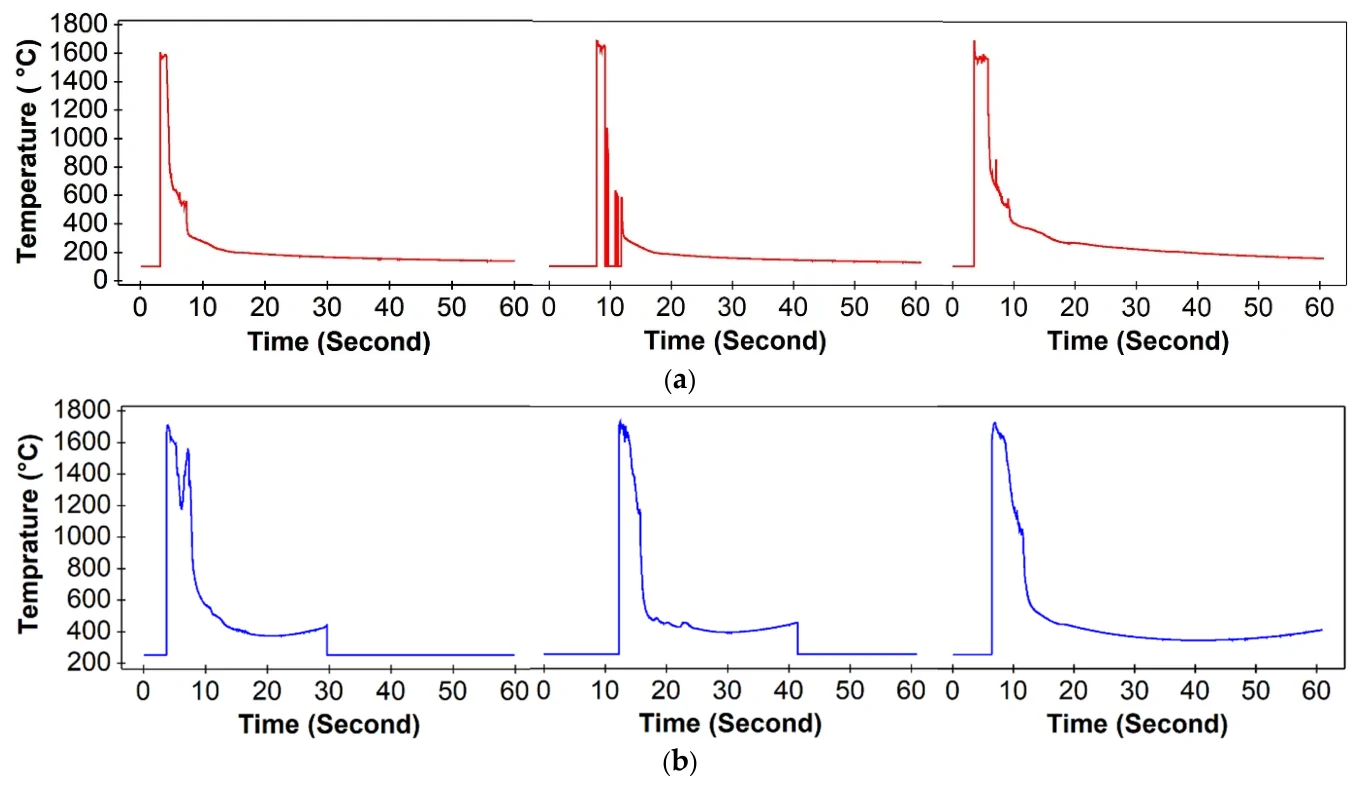
Surface temperature of the best-cut plasma surface using the (**a**) mixture of oxygen and compressed air and (**b**) compressed air only.

Figure 18 depicts the temperature profile recorded for the best-cut plasma surface using the mixture of oxygen and compressed air (Figure 18a) and the best-cut plasma surface using compressed air (Figure 18b) only. It was observed that the temperature profile in the case of the plasma-cut surface using the mixture of oxygen and compressed air was very stiff and cooled relatively slowly compared to the plasma surface using compressed air only. This is also reflected in the obtained slow cooling rates for the plasma-cut surface using the mixture of oxygen and compressed air (Figure 7c, Figure 8c and Figure 9c). The addition of oxygen with compressed air helped in the oxidation of iron atoms and releases a high degree of energy (267–412 kJ/mol), which is additional energy for the process, along with the energy of the focused plasma arc. On average, the amount of energy supplied by the burning reaction is about 60% for mild steel [29]. The balanced chemical equations for the exothermic reaction of the mild steel are as follows:

(1) Fe+O →FeO+267 kJ of heat generation 

(2) $$3Fe+{2O}_{2}\;\to {Fe}_{3}O_{4}+1120\;\mathrm{kJ}\;of\;heat\;generation$$

(3) $$2Fe+1.5O_{2}\;\to {Fe}_{2}O_{3}+825\;\mathrm{kJ}\;of\;heat\;generation$$

(4) $$C+O_{2\;}\to CO_{2}+393\;\mathrm{kJ}\;of\;heat\;generation$$

(5) $$Mn+O_{2\;}\to MnO_{2}+521\;\mathrm{kJ}\;of\;heat\;generation$$

It helps to establish stable and high-energy plasma at low amperes for cutting work materials, which indirectly reduces surface temperature and the heat-affected zone.

### 3.7. Assessment of Process Efficiency

**Figure 19:**
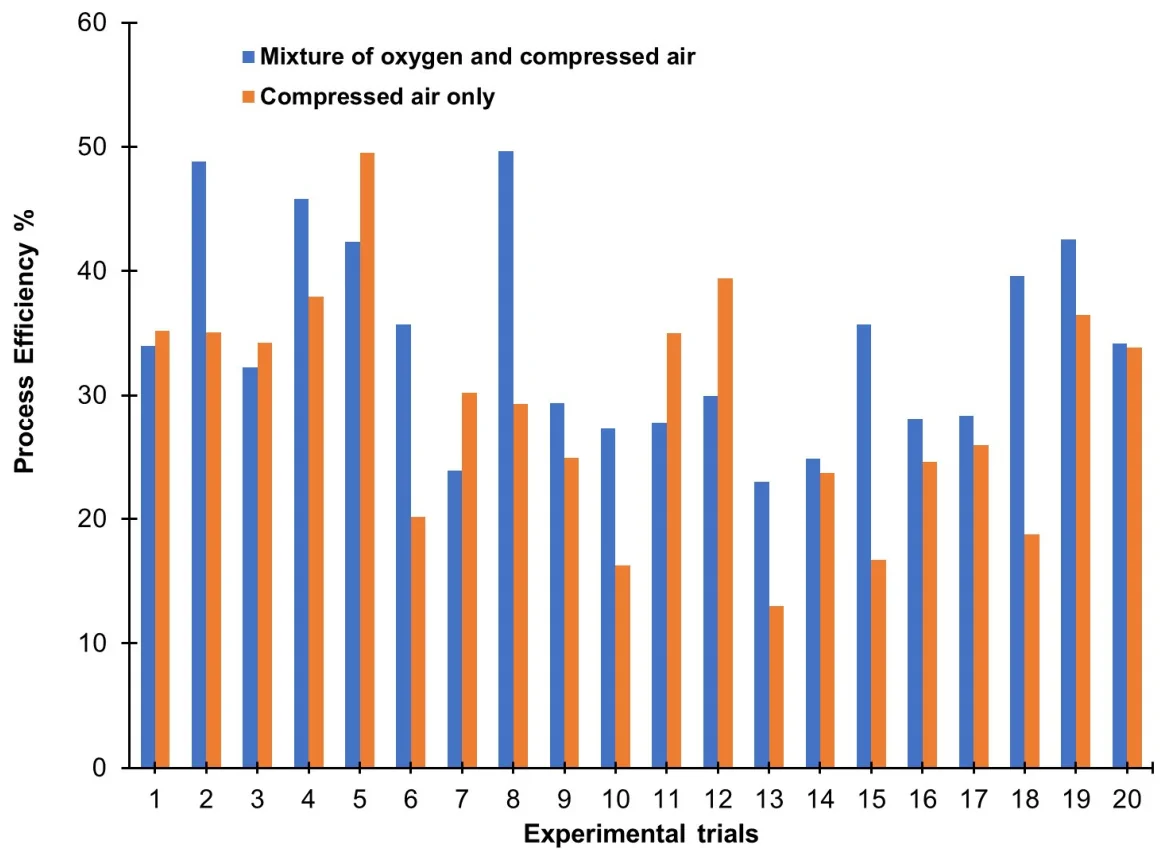
Plasma cutting process efficiency using a mixture of oxygen and compressed air, and compressed air only.

Figure 19 depicts the process efficiency of the PAC process while using a mixture of oxygen and compressed air and compressed air alone as the carrier medium. The process efficiency of the plasma cutting process (η) is defined as the ratio of thermal energy employed for melting and removing materials from the kerf to the total plasma power supplied by the plasma torch. The following relation was used to compute the process efficiency [30]:

(6) $$\eta =\frac{m[C_{p}\left(T_{s}-T_{r}\right)+\;L_{f}]}{V_{a\;}I}$$

where *m* is material removal rate (i.e., ρ K_w_ *Vt*) (g/s), ρ is the density of low carbon steel (≈7.85 × 10^6^ g/m^3^), K_w_ is the kerf width (m), *V* is the cutting speed (m/s), t is the thickness of plate (m), *C_p_* is the specific heat capacity of low carbon steel (≈0.49 j/g°C), *T_a_* is the Surface temperature (°C), *T_r_* is the room temperature (i.e., 27 °C), and *L_f_* is the latent heat of fusion for low-carbon steel (≈270 J/g). The power source used in these experiments has the facility to vary current only; therefore, plasma power is calculated in terms of multiples of current and voltage. Therefore, an average voltage is taken as 125 V for the calculation of process efficiency [9,31].

It is evident from the figure that the process efficiency of the compressed air-containing plasma cutting process is very high during the initial phases of experimental trials, as compared to the oxygen and compressed air-containing plasma cutting process. Thereafter, it is nearly as close to the process efficiency of oxygen and compressed air-containing plasma cutting, and it then starts dropping further. The overall process efficiency of oxygen and compressed air-containing plasma cutting was found to be higher than that of compressed air cutting. This is because mixing oxygen with compressed air causes an exothermic reaction and raises the plasma heat content, which further improves cutting capacity and increases the process efficiency.

Plasma air cutting is a highly efficient, versatile, and accessible thermal cutting process widely used in fabrication, automotive repair, and industrial manufacturing. It could also be automated using different types of CNC systems for applications to cut a variety of shapes, ranging from simple straight lines to circles to complex profiles. It uses compressed air as a carrier medium. However, the experimental study reveals that the addition of oxygen improved the cut surface quality, surface finish, morphology, and microhardness of the cut surface. This could be because the use of compressed air alone has a lower plasma-blowing capacity or requires a higher shearing force, causing an increase in heat content or a low cooling rate, which in turn deteriorates the cut-surface quality and forms a larger range of HAZ. In case of cutting with oxygen and compressed air plasma flame, the addition of oxygen enhances the speed of the plasma flame towards the target, and the exothermic reaction of oxygen with the parent metal causes an increase in heat content, which significantly increases plasma cutting capacity and produces good cut-surface quality. The rise in cooling rate helps in atomic diffusion and develops soft, coarse, and less dislocated phases of ferrite structure, which makes post-processing of metal easier with less material removal. The striation depth in the case of oxygen and compressed air-assisted plasma cutting was greater compared to compressed air-assisted plasma cutting due to the exothermic reaction of oxygen, which raises plasma heat content and reduces solidification over the cut surface and improves its quality.

## 4. Conclusions

This paper reports an experimental study on oxygen-assisted plasma air cutting and its comparison with compressed-air plasma cutting. The following conclusions can be drawn from this study:

- The heat content for penetration of work material increases with a rise in plasma current. Use of excessive current value may lead to more material removal, which enhances kerf width, dross height, bevel angle, surface roughness and localised heating, causing more HAZ. RSM optimisation suggests a correlation between plasma current and kerf width, dross height, bevel angle, surface roughness and HAZ.
- The rise in cutting speed reduces heat concentration on a given area and increases cooling time, which results in a reduction in the values of considered responses. Excessive rise in cutting speed gives incomplete penetration and results in excessive dross with poor kerf width, bevel angle, and high surface roughness values. Cutting speed demonstrated a non-linear quadratic relationship that affects the roughness of the surface.
- The addition of oxygen improved plasma stability and blown capacity, which gives a fine kerf width, lower bevel angle, and dross height. The improved blown capacity removed dislodged materials from the kerf, which reduces surface roughness, surface temperature and HAZ. This acts as a significant factor that shifts the performance, statistically minimising variance and lowering the mean values of surface roughness, bevel angle, and HAZ. There is a strong statistical interaction effect between gas composition and plasma current. The data proves that adding oxygen allows the plasma to stabilise at a low current while maintaining efficiency.
- A surface morphology study of the best finished plasma-cut surface revealed that the addition of oxygen improved surface quality. Microstructural analysis confirms a melt-and-blast thermal–mechanical mechanism characterised by grain coarsening, phase transformations, and thermal erosion from oxygen acceleration.
- The reduced values of microhardness will improve the machinability of work materials. The addition of oxygen with compressed air stabilised the high-energy plasma at a low plasma current, which reduced the heat-affected zone and improved the post-processing work of cut-surface materials.
- The addition of oxygen with compressed air improved plasma process efficiency, but it reduced nozzle life significantly because of a rise in plasma temperature.
- This work shows that the addition of oxygen along with compressed air improved plasma-cut surface quality in terms of kerf width, bevel angle, dross generation, surface finish and heat-affected zone with a low value of plasma current. The outcome of this study will be very helpful for the industries and tool rooms that frequently use the plasma cutting process for sheet-metal cutting work and for post-processing of work materials for manufacturing various industrial components.

## Figures and Tables

**Table 1:** Values of optimum variable parameters and responses for the best-cut plasma surface using compressed air.

Optimum Variable Input Parameters			Responses						
Current ‘I’ (A)	Cutting speed ‘V’ (m/min)	Torch Stand-off distance ‘*d*’ (mm)	Kerf width ‘K_w_’ (mm)	Dross height ‘d_h_’ (mm)	Bevel angle ‘α’ (Radian)	Avg. surface roughness ‘R_a_’ (µm)	Max. surface roughness ‘R_max_’ (µm)	Max. surface temperature ‘T_s_’ (°C)	HAZ ‘H_z_’ (µm)
80	0.8	5	2.3	0.3	1.6	1.2	8.2	1719.3	38

**Table 2:** Identified values of variable and fixed input parameters used during experiments.

Variable Input Parameter with Its Symbol (Unit)	Levels				
	(−2)	(−1)	0	(+1)	(+2)
Current ‘I’ (A)	50	60	70	80	90
Cutting speed ‘V’ (m/min)	0.5	0.6	0.7	0.8	0.9
Oxygen gas contribution ‘C_g_’ (%) (i.e., Inlet gas pressure ‘P_I_’ = oxygen pressure ‘P_o_’ + compressed air pressure ‘P_c_’)	40 (2.8 + 4.2)	50 (3.5 + 3.5)	60 (4.2 + 2.8)	70 (4.9 + 2.1)	80 (5.6 + 1.4)

Fixed input parameters: Stand-off distance ‘d’: 4 mm, Cut length ‘l’: 30 mm.

**Table 3:** Parameters and the considered responses for oxygen-assisted plasma air cutting of C-20 carbon steel during experiments.

Exp. No.	Variable Input Parameters			Responses						
	Current ‘I’ (A)	Cutting Speed ‘V’ (m/min)	Oxygen Gas Contribution ‘C_g_’ (%)	Kerf Width ‘K_w_’ (mm)	Dross Height ‘d_h_’ (mm)	Bevel Angle ‘α’ (Radian)	Avg. Surface Roughness ‘R_a_’ (µm)	Max. Surface Roughness ‘R_max_’ (µm)	Max. Surface Temperature ‘T_s_’ (°C)	HAZ ‘H_z_’ (µm)
1	60	0.6	70	2.7	1.6	1.5	0.8	5.3	1931.7	53.6
2	70	0.7	80	2.8	2.4	1.6	1.2	6.5	1963.0	71.0
3	80	0.6	50	3	2.7	1.6	1.3	7.5	1910.0	71.6
4	80	0.6	70	3	2.7	1.6	1.4	8.0	1932.0	72.1
5	60	0.6	50	2.6	1.4	1.5	0.8	5.1	1893.5	52.1
6	70	0.7	60	2.1	0.6	1.4	0.6	2.5	1689.9	37.9
7	50	0.7	60	2.7	1.7	1.5	1.0	5.7	1993.8	60.9
8	70	0.9	60	3	2.5	1.6	1.3	7.3	1987.0	71.4
9	70	0.7	60	2.5	1.1	1.5	0.5	4.0	1767.1	46.9
10	60	0.8	50	2.7	1.6	1.5	0.9	5.3	1957.0	58.6
11	70	0.7	60	2.4	0.8	1.5	0.4	2.6	1692.3	44.6
12	70	0.7	60	2.4	1.0	1.5	0.5	3.0	1726.8	45.7
13	70	0.7	60	2.5	1.2	1.5	0.7	4.0	1810.7	47.7
14	70	0.7	60	2.1	0.2	1.4	0.2	1.6	1607.8	23.2
15	70	0.5	60	2.7	1.8	1.5	1.0	6.0	1985.0	61.0
16	80	0.8	70	3.1	3.2	1.6	1.9	8.0	1968.0	74.0
17	90	0.7	60	3.4	3.7	1.6	2.2	15.0	1996.0	84.3
18	80	0.8	50	3.3	3.3	1.6	2.2	10.0	1989.0	78.0
19	70	0.7	40	2.8	2.3	1.6	1.0	6.2	1962.0	61.3
20	60	0.8	70	2.6	1.4	1.5	0.7	4.2	1860.1	48.3

**Table 4:** Kerf width, dross height, and bevel angle of the best-cut plasma surface using the mixture of oxygen and compressed air and the best-cut plasma surface using compressed air.

Response Name (Unit)	Values Using the Mixture of Oxygen and Compressed Air	Values Using Compressed Air
Kerf width ‘K_w_’ (mm)	2.1	2.3
Dross height ‘d_h_’ (mm)	0.2	0.3
Bevel angle ‘α’ (Radian)	1.4	1.6

## Data Availability

The original contributions presented in this study are included in the article/Supplementary Material. Further inquiries can be directed to the corresponding authors.

## Supplementary Materials

The following supporting information can be downloaded at: https://www.mdpi.com/article/10.3390/ma19204293/s1, Table S1: Analysis of variance (ANOVA) table for responses; Table S2: Optimized values of Plasma Parameters and Responses using Desirability Function Analysis.
