# Supplementary material for: Comparative Evaluation of Oxygen-Assisted and Compressed-Air Plasma Cutting of Low-Carbon Steel
Source: Materials (Basel). 2026 Oct 12;19(20):4293. doi: 10.3390/ma19204293 (PMC13706544; doi:10.3390/ma19204293)
Supplement: Supplementary file 1 [file materials-19-04293-s001.zip › materials-4512806-supplementary.pdf]

Table S1. Analysis of variance (ANOVA) table for responses

Source	Sum of Squares	df	Mean Square	F-Value	P-value	Remark
For Kerf width						
Model	2.03	9	0.23	8.53	0.0012	*significant*
A-Current	*0.68*	*1*	*0.68*	*25.78*	*0.0005*	
B-Cutting speed	*0.076*	*1*	*0.076*	*2.86*	*0.1214*	
C-Oxygen gas contribution	*6.250E-004*	*1*	*6.250E-004*	*0.024*	*0.8808*	
AB	*0.011*	*1*	*0.011*	*0.43*	*0.5286*	
AC	*0.011*	*1*	*0.011*	*0.43*	*0.5286*	
BC	*0.011*	*1*	*0.011*	*0.43*	*0.5286*	
A^2	*0.81*	*1*	*0.81*	*30.70*	*0.0002*	
B^2	*0.51*	*1*	*0.51*	*19.21*	*0.0014*	
C^2	*0.42*	*1*	*0.42*	*15.98*	*0.0025*	
Residual	0.26	10	0.026			
Lack of Fit	*0.091*	*5*	*0.018*	*0.52*	*0.7528*	*not significant*
Pure Error	*0.17*	*5*	*0.035*			
Cor Total	17.35	19				
R^2	0.8848					
**Equation in terms of coded factor for Kerf width** $= 2.36 + 0.41A + 0.14B - 0.012C + 0.15A B -0.15AC -0.15BC +0.72A^2 +0.57 B^2 +0.52C^2$						
For dross height						
Model	16.19	9	1.80	15.45	< 0.0001	*significant*
A-Current	*6.45*	*1*	*6.45*	*55.42*	*< 0.0001*	
B-Cutting speed	*0.49*	*1*	*0.49*	*4.21*	*0.0673*	
C-Oxygen gas contribution	*1.000E-002*	*1*	*1.000E-002*	*0.086*	*0.7754*	
AB	*0.080*	*1*	*0.080*	*0.69*	*0.4265*	
AC	*0.020*	*1*	*0.020*	*0.17*	*0.6873*	
BC	*5.000E-003*	*1*	*5.000E-003*	*0.043*	*0.8400*	
A^2	*5.40*	*1*	*5.40*	*46.43*	*< 0.0001*	
B^2	*3.28*	*1*	*3.28*	*28.17*	*0.0003*	
C^2	*4.25*	*1*	*4.25*	*36.51*	*0.0001*	
Residual	1.16	10	0.12			
Lack of Fit	*0.48*	*5*	*0.095*	*0.69*	*0.6524*	*not significant*
Pure Error	*0.69*	*5*	*0.14*			
Cor Total	17.35	19				
R^2	0.9329					
**Equation in terms of coded factor for Dross height** $= 0.87+1.27A+0.35B+0.050C+0.40AB-0.20\ AC-0.10BC+1.85A^2+1.44\ B^2+1.64C^2$						
**For Bevel angle**						
Model	0.063	9	7.040E-003	3.78	0.0250	*significant*
A-Current	*0.022*	*1*	*0.022*	*12.07*	*0.0060*	
B-Cutting speed	*2.500E-003*	*1*	*2.500E-003*	*1.34*	*0.2737*	
C-Oxygen gas contribution	*0.000*	*1*	*0.000*	*0.000*	*1.0000*	
AB	*0.000*	*1*	*0.000*	*0.000*	*1.0000*	
AC	*0.000*	*1*	*0.000*	*0.000*	*1.0000*	

BC	*0.000*	*1*	*0.000*	*0.000*	*1.0000*	
A^2	*0.012*	*1*	*0.012*	*6.29*	*0.0310*	
B^2	*0.012*	*1*	*0.012*	*6.29*	*0.0310*	
C^2	*0.029*	*1*	*0.029*	*15.68*	*0.0027*	
Residual	0.019	10	1.864E-003			
Lack of Fit	*5.303E-003*	*5*	*1.061E-003*	*0.40*	*0.8328*	*not significant*
Pure Error	*0.013*	*5*	*2.667E-003*			
Cor Total	0.082	19				
R^2	0.7727					
**Equation in terms of coded factor for Bevel angle** =1.47+0.075A+0.025B+0.000C+0.000AB+0.000 AC+0.000BC+0.086A²+0.086B²+0.14C²						
**For Avg. Surface roughness**						
Model	5.62	9	0.62	13.41	0.0002	*significant*
A-Current	*2.59*	*1*	*2.59*	*55.70*	*< 0.0001*	
B-Cutting speed	*0.29*	*1*	*0.29*	*6.27*	*0.0313*	
C-Oxygen gas contribution	*7.225E-003*	*1*	*7.225E-003*	*0.16*	*0.7018*	
AB	*0.14*	*1*	*0.14*	*2.91*	*0.1191*	
AC	*0.016*	*1*	*0.016*	*0.35*	*0.5683*	
BC	*0.011*	*1*	*0.011*	*0.24*	*0.6336*	
A^2	*1.87*	*1*	*1.87*	*40.09*	*< 0.0001*	
B^2	*0.85*	*1*	*0.85*	*18.22*	*0.0016*	
C^2	*0.84*	*1*	*0.8 4*	*17.97*	*0.0017*	
Residual	0.47	10	0.047			
Lack of Fit	*0.33*	*5*	*0.066*	*2.40*	*0.1792*	*not significant*
Pure Error	*0.14*	*5*	*0.027*			
Cor Total	6.08	19				
R^2	0.9235					
**Equation in terms of coded factor for Avg. Surface roughness** =0.56+0.81A+0.27B+0.043C+0.52AB-0.18AC-0.15BC+1.09A²+0.73B²+0.73C²						
**For Max. Surface roughnness**						
Model	162.75	9	18.08	14.82	0.0001	*significant*
A-Current	*71.32*	*1*	*71.32*	*58.47*	*< 0.0001*	
B-Cutting speed	*2.00*	*1*	*2.00*	*1.64*	*0.2290*	
C-Oxygen gas contribution	*9.025E-003*	*1*	*9.025E-003*	*7.399E-003*	*0.9332*	
AB	*0.46*	*1*	*0.46*	*0.38*	*0.5525*	
AC	*0.53*	*1*	*0.53*	*0.43*	*0.5245*	
C	*0.58*	*1*	*0.58*	*0.48*	*0.5050*	
A^2	*73.33*	*1*	*73.33*	*60.12*	*< 0.0001*	
B^2	*23.61*	*1*	*23.61*	*19.36*	*0.0013*	
C^2	*20.27*	*1*	*20.27*	*16.62*	*0.0022*	
Residual	12.20	10	1.22			
Lack of Fit	*7.86*	*5*	*1.57*	*1.81*	*0.2648*	*not significant*
Pure Error	*4.34*	*5*	*0.87*			
Cor Total	174.95	19				
R^2	0.9303					
**Equation in terms of coded factor for Max. Surface roughness** = 3.08 +4.22A +0.71B -0.047C +0.96AB -1.03AC -1.08BC +6.83A² +3.88B²+3.59C²						
**For Max. Temperature**						
Model	3.004E+005	9	33377.45	7.99	0.0016	*significant*

A-Current	*17808.90*	*1*	*17808.90*	*4.26*	*0.0659*	
B-Cutting speed	*985.96*	*1*	*985.96*	*0.24*	*0.6376*	
C-Oxygen gas contribution	*351.56*	*1*	*351.56*	*0.084*	*0.7777*	
AB	*1971.92*	*1*	*1971.92*	*0.47*	*0.5077*	
AC	*703.13*	*1*	*703.13*	*0.17*	*0.6903*	
BC	*0.24*	*1*	*0.24*	*5.864E-005*	*0.9940*	
A^2	*89440.92*	*1*	*89440.92*	*21.41*	*0.0009*	
B^2	*1.520E+005*	*1*	*1.520E+005*	*36.38*	*0.0001*	
C^2	*1.520E+005*	*1*	*1.520E+005*	*36.38*	*0.0001*	
Residual	41782.29	10	4178.23			
Lack of Fit	*17136.53*	*5*	*3427.31*	*0.70*	*0.6501*	*not significant*
Pure Error	*24645.75*	*5*	*4929.15*			
Cor Total	3.422E+005	19				
R^2	0.8779					
**Equation in terms of coded factor for Max. Temperature** =1724.66+36.85A+30.57B+10.00C-5.30AB-37.00AC-22.20BC+230.07A²+288.02B²+264.02C²						
**For Heat affected zone (HAZ)**						
Model	2.382E+005	9	26466.71	6.16	0.0044	*significant*
A-Current	*5431.69*	*1*	*5431.69*	*1.26*	*0.2873*	
B-Cutting speed	*3739.32*	*1*	*3739.32*	*0.87*	*0.3730*	
C-Oxygen gas contribution	*400.00*	*1*	*400.00*	*0.093*	*0.7666*	
AB	*14.04*	*1*	*14.04*	*3.267E-003*	*0.9555*	
AC	*684.50*	*1*	*684.50*	*0.16*	*0.6983*	
BC	*246.42*	*1*	*246.42*	*0.057*	*0.8156*	
A^2	*83181.15*	*1*	*83181.15*	*19.35*	*0.0013*	
B^2	*1.304E+005*	*1*	*1.304E+005*	*30.32*	*0.0003*	
C^2	*1.095E+005*	*1*	*1.095E+005*	*25.48*	*0.0005*	
Residual	42996.90	10	4299.69			
Lack of Fit	*18351.15*	*5*	*3670.23*	*0.74*	*0.6229*	*not significant*
Pure Error	*24645.75*	*5*	*4929.15*			
Cor Total	2.812E+005	19				
R^2	0.8471					
**Equation in terms of coded factor for Heat affected zone (HAZ)** =41.99+17.81A+5.38B+2.45C-2.82AB-3.74AC-1.91B C+27.19A²+27.08B²+27.04C²						

Table S2. Optimized values of Plasma Parameters and Responses using Desirability Function Analysis

**Current 'I' (A)**	**Cutting speed 'V' (m/min)**	**Oxygen gas contribution 'C_g' (%)**	**Kerf width** 'K_w' **(mm)**	**Dross height** 'd_h' **(mm)**	**Bevel angle** 'α' **(Radian)**	**Avg. surface roughness 'R_a'(μm)**	**Max. surface roughness 'R_{max}'(μm)**	**Max. surface temperature 'T_s' (°C)**	**HAZ 'H_z' (μm)**	**Overall Desirability**
64.22	6.83	59.33	2.29	0.641	1.45	0.41	2.41	1731.38	38.65	0.820
